# Ultralow loss visible light metamaterials assembled by metaclusters

**DOI:** 10.1515/nanoph-2022-0171

**Published:** 2022-05-13

**Authors:** Jing Zhao, Huan Chen, Kun Song, Liqin Xiang, Qian Zhao, Chaohong Shang, Xiaonong Wang, Zhijie Shen, Xianfeng Wu, Yajie Hu, Xiaopeng Zhao

**Affiliations:** Medtronic plc, Boulder, CO 80301, USA; Smart Materials Laboratory, Department of Applied Physics, Northwestern Polytechnical University, Xi’an 710129, P. R. China; State Key Lab Tribology, Department of Mechanical Engineering, Tsinghua University, Beijing 100084, P. R. China

**Keywords:** 3D negative index metamaterials, inverse Doppler effect, isotropic clusters, ultralow loss, visible spectrum

## Abstract

Optical metamaterials give birth to the control and regulation of light. However, because of strong energy dissipation and fabrication difficulty in meta-atoms, low-loss isotropic three dimensional negative index metamaterials (NIMs) in the visible spectrum has long been regarded as an extremely challenging. Here, we report an ultralow loss isotropic metamaterials for visible light and its inverse Doppler effect. The ball-thorn-shaped metaclusters with symmetrical structure consisting of the dielectric and its surface dispersed super-thin silver layer was proposed, the surface plasma resonance is formed by discrete silver layer with a thickness of two or three atomic layers. We invented a unique technique for preparing ultralow loss isotropic clusters and three-dimensional large block samples. The negative refractive index and the inverse Doppler effect of green and red light is measured by the prism method for the first time. The discrete super-thin silver layer produced by the photoreduction method greatly reduces the generation of loss and break through noble metal high energy losses of traditional optical frequency metamaterial, the metaclusters unfold bottleneck of the nano-assemble visible light metamaterials, opening a door for disorder assembling ultralow loss isotropic three-dimensional large block NIMs devices of arbitrary shape.

## 1 Introduction

The experimental verification of negative refraction embarked on a new wave of innovation in metamaterials [1], [2], [3], [4], [5]. Metamaterials are artificial media made of subwavelength metallic and dielectric periodic structure with properties that do not present in natural materials [1, 6, 7]. Metallic wires and split-rings (meta-atoms) were identified as building blocks to realize negative refraction by Pendry in his pioneering works [8, 9]. Extraordinary electromagnetic properties in the microwave range associated with negative-index metamaterials (NIMs), including super-resolution lens [6], electromagnetic cloaking [7], and inverse Doppler effect [10], [11], [12] have been reported over the last two decades.

Optical NIMs particularly in visible light make it possible to manipulate light at subwavelength scale [2, 13]. Because of strong energy dissipation and significant fabrication difficulty in metals [14, 15], low-loss isotropic three dimentional NIMs in the visible spectrum has long been regarded as an extremely challenging difficulty [3, 16, 17]. As a result, mainstream fabrication methods for optical metamaterials nowadays employ various etching techniques to forms meta-atoms [15], such as the double-fishnet [17, 18] and the 3D wedge-shaped fishnet [16, 19]. However, the required structural features for optical negative-index metamaterials such as a metallic nanogap and complicated 3D networks would be difficult to achieve with conventional monolithic lithography [20]. Self-assembled colloidal soft materials have been extensively studied to pursue the achievement of unnatural refractive index at optical frequencies [20], [21], [22], [23]. Self-assembled colloidal soft materials have emerged as a new platform for the preparation of optical metamaterials, which can promote the massive and large-scale applications of optical metamaterials and accelerate development of tunable optical devices with unprecedented functionality and performance characteristics [24]. The relevant works provide advances in the synthesis of metallic [25, 26] and high-index dielectric [27, 28] colloids, and explore the properties of them in different experimental protocols, including meta-fluid [29], assembling them into metaclusters [30], [31], [32], [33], [34] and condensed films [35, 36]. Unfortunately, although an unnaturally high refractive index has been experimentally verified [28, 36], these works were unable to reach the negative refractive index. Much progress has been made in recent years with respect to developing bottom–up preparation approaches for metamaterials [37], [38], [39], [40], but metamaterials based on the concept of noble metallic meta-atoms structure suffer from many inherent performance limitations including considerable Ohmic loss, optical anisotropy, and difficulty in tuning [13, 14]. The Mie resonances of dielectric particles provide a possible mechanism for realizing magnetic or electric resonance. Thanks to dielectric sphere’s structure simplicity and isotropy, Mie resonance-based microwave all-dielectric metamaterials have already been realized in experimental settings [41, 42], suggesting a promising direction for optical metamaterials [43, 44]. Yet, this approach demands high permittivity for dielectric particles to generate strong resonant electromagnetic fields for loss minimization, resulting in a practically difficult route to engineer isotropic infrared and visible light metamaterials. Metasurfaces that control light waves by introducing an abrupt phase shift at subwavelength scale have been proposed as an alternative approach [45, 46]. Nevertheless, limited successes in the visible spectrum have been achieved to date [5]. Although plasmonic materials with a lower loss than noble metals have long been sought, the stable sodium-based plasmonic devices with state-of-the-art performance at near-infrared wavelengths were not available until quite recently [3]. Recently, by engineering of the dispersion properties of a photonic crystal (PhC), the photonic bands for 3D PhCs capable of negative refraction in the mid-infrared was described [4].

Loss at the resonance frequency sometimes severely impairs metamaterial’s extraordinary performance [2, 13]. This problem becomes more prominent for the visible light because its skin depth is comparable to the thickness of metallic traces that are commonly found in a metamaterial unit cell [3, 14]. Noble metals such as silver and gold are primary candidates for engineering frequency selective materials at optical frequencies. Published designs including the metal–dielectric–metal fishnet structures consist of a single functional layer along the direction of propagation [17, 18], the 3D optical NIMs made of cascaded fishnet metamaterial [15, 16, 19] are all based on the meta-atoms which results in an optimal metal thickness of cell unit being about 20–50 nm. As a result, considerable loss is induced by volumetric currents and plasma resonances, making metamaterial properties much less attractive for meaningful applications. Up to this day, a significant scientific breakthrough in the visible zero-loss negative-index 3D isotropic metamaterials remain to be seen [3, 13, 44].

Here, we demonstrate the first ultralow loss isotropic metamaterials in the visible spectrum. The ball-thorn-shaped meta-clusters with symmetrical structure consisting of the dielectric and its surface dispersed super-thin silver layer have replaced the lithographically defined meta-atoms in existing NIMs, it is found that the discrete super-thin silver layer produced by the photoreduction method can stimulate the surface plasmon resonance required for metamaterials. The unique technique for preparing ultralow loss isotropic clusters and three-dimensional large block samples by a disorder assemble approach was invented. Using the prism method, we report the negative refractive index and the inverse Doppler effect of green and red light in experiment for the very first time. The proposed ball-thorn-shaped metaclusters structure break through noble metal high energy losses of traditional optical frequency metamaterial and difficult to achieve dielectric property constraints of all-dielectric metamaterial, opening a door for disorder assembling ultralow loss isotropic three-dimensional large block NIMs devices of arbitrary shape.

## 2 Meta-clusters structure and property

### 2.1 Design and behavior of the metaclusters structure

**Figure 1:**
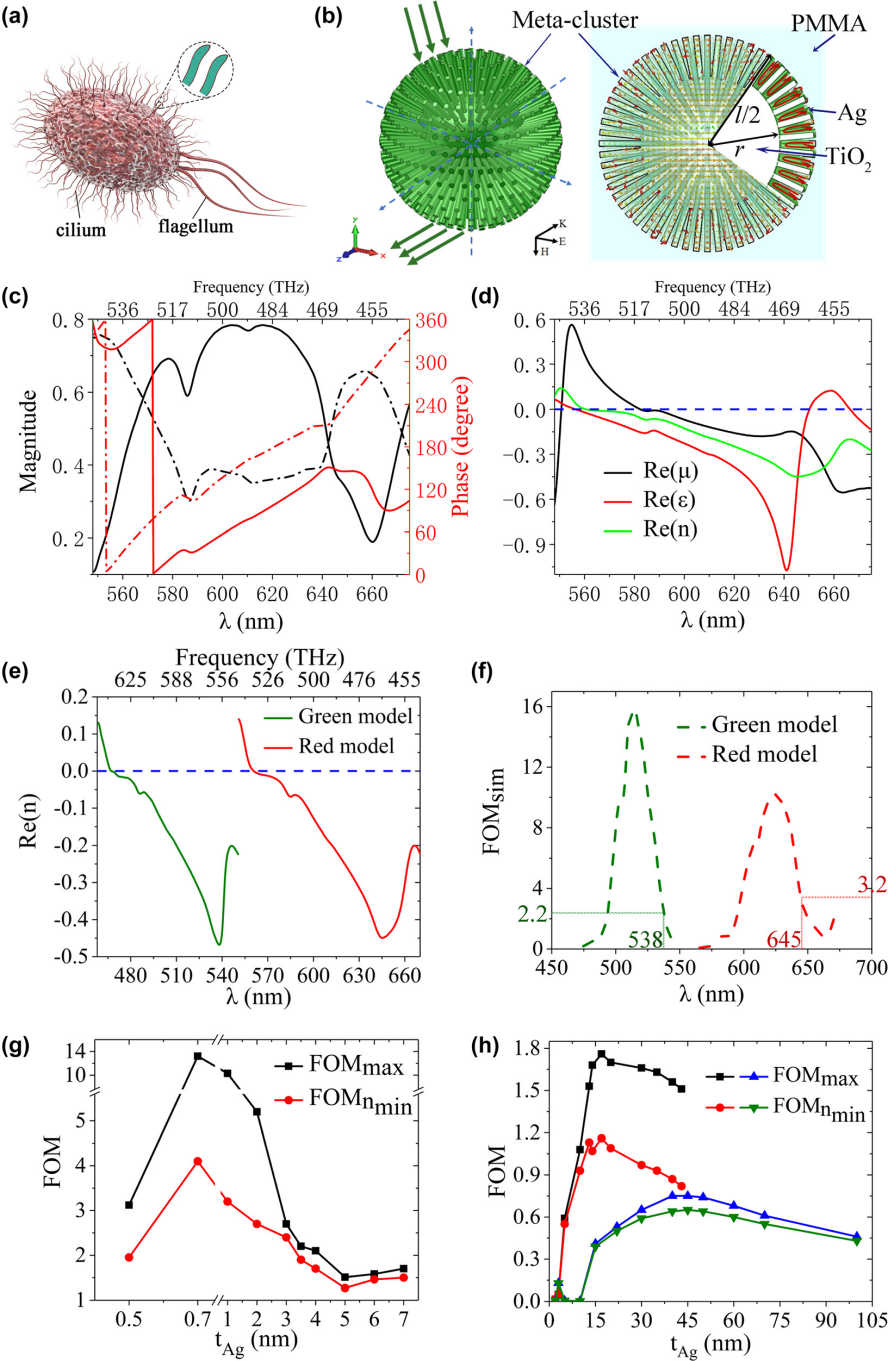
Behavior of the metaclusters structure. (a) Schematic of the biological cilium-cell. (b) Green light wave band metacluster model (left Figure), the cluster is composed of a spherical core and many prominent rods. When the response band light ray incident, the meta-clusters will occur negative refraction effect. The profile current distribution perpendicular to the external magnetic field and the 1/8 model profile (right picture). The protruding rods and epidermic connections form “U” shaped effective units, the negative permeability is generated when the annular current and the external magnetic field interact with each other, which is regarded as the basic effective units of the meta-cluster (there are ∼1200 units). The whole meta-cluster is in the PMMA environment background. The model profile shows that the model is filled with TiO_2_ medium (white area) and the epidermis is covered with a very thin layer of Ag (green part, 1 nm). (c) Transmission (solid line) and reflection (dot-dash line) coefficient for the red-light metaclusters with *l* = 640 nm, *r* = 215 nm, and *P* = 670 nm. (d) The effective parameters for the red-light metaclusters retrieved from the coefficients in (c). (e) Effective refractive indices for the red-light metaclusters (red line) and the green-light metaclusters (green line) with *l* = 530 nm, *r* = 165 nm, and *P* = 560 nm. (f) FOM curves of the metaclusters resonating at the red-light (red dotted line) and green-light (green dotted line), respectively. (g) FOM of the red-light metaclusters structure as a function of Ag layer thickness *t*_Ag_. (h) FOMs of fishnet structures at different Ag layer thickness. Black square and red circle lines represents the results obtained using the Ag–Al_2_O_3_–Ag fishnet structure whose geometrical parameters refer to the published work [17] and blue triangle and green inverted-triangle lines represents the results obtained using the Ag–MgF_2_–Ag fishnet structure whose geometrical parameters refer to the published work [19].

It is known that biological cells are the basic building blocks of all organisms. Cilia, consisting of internal cytoplasm and surface plasmalemma, can be found on the surface of a cell (see Figure 1a). Cilia are known for their importance as the ‘antennas’ of a cell and their functions in terms of stimulating responses to surrounding environment, which include chemical sensation, signal transduction, and control of cell growth. Inspired by the ciliated cell structure, we created a ball-thorn-shaped metamaterial cluster (metacluster) model consists of a spherical kernel and many protruding rods (Figure 1b, left picture) as analogous to the cilium-cell structure found in nature. Both the kernel and rods are made of TiO_2_ coated by Ag of 1 nm in thickness. 600 identical rods with cross-sectional diameter of 15 nm are uniformly distributed around the surface of a kernel. *l* represents the diameter of the meta-cluster, *r* is the radius of the spherical kernel, and *P* refers to the lattice constant of the meta-cluster, the meta-cluster is fully immersed in polymethyl methacrylate (PMMA). Through a lot of computational selection and optimization, we chose the aspect ratio as described above. In fact, each ball-thorn-shaped meta-cluster can be regarded as composed of 1200 meta-atoms: U-shaped split-ring [9] and equivalent wires [8] distributed evenly and symmetrically along the space. These meta-atoms geometrical size is much smaller than the wavelength, and it is their independent resonance that forms the meta-cluster resonant response outfield (Figure 1b, right picture).

This meta-cluster model is solved in Computer Simulation Technology (CST) Microwave Studio (Supplementary Information S1). The relative permittivity of Ag is set to be consistent with the actual Drude model value [47], of TiO_2_ is 5.2 with a dissipation factor of 0.003, of PMMA is 2.5. A peak in transmission coefficient indicates the meta-cluster resonates within the wavelength range of the red-light (Figure 1c), a Mie resonance [41, 48]. For convenience of comparison with experiments, only the results of the first order structure are presented here. The effective parameters prove that the material composed of this structure is a metamaterial (Figure 1d). At λ = 645 nm, the value of Re(*n*) reaches a minimum of −0.45 (Figure 1e). The figure of merit (FOM) curve (in red) of the meta-cluster in the red-light band is shown in Figure 1f, where FOM_sim_ = −Re(*n*)/Im(*n*) for Re(*n*) < 0, and Re(*n*) and Im(*n*) are the real and imaginary parts of the refractive index, respectively. FOM_sim_ arrives at a maximum of 10.3 at λ = 623 nm and is about 3.2 at λ = 645 nm where the value of Re(*n*) is the most negative. To achieve a similar effect in the green-light band, we reduced *l* = 530 nm, *r* = 165 nm, *P* = 560 nm. As expected, the transmission and reflection curves indeed reveal a Mie resonance at the green-light wavelengths (Figure S1). Similarly, the permeability, permittivity, and refractive index are simultaneously negative at near 530 nm. At λ = 538 nm, the value of Re(*n*) reaches a minimum of −0.47 (Figure 1e). The FOM curve (in green) of the green-light meta-cluster is shown in Figure 1f. FOM_sim_ arrives at a maximum of 15.9 at λ = 514.5 nm and is about 2.2 at λ = 538 nm where the value of Re(*n*) is the most negative.

Based on effect of Ag layer thickness *t*_Ag_ on the response behavior of the red-light metaclusters in PMMA medium (Figure S5), Figure 1g shows the relationship between metal film thickness and FOM variation. It can be seen that with silver as resonant material, the plasma resonance can be formed with a height of only two or three atomic layers, resulting in an optimized silver coating thickness being about 1 nm. However, in the models of meta-atom cell unit, such as double fishing nets [18, 19] or nanowires [40], the optimal metal film thickness of the unit is 20–50 nm (Figure 1h). Our cluster design is independent of the previously widely used meta-atom cell design, this model greatly reduces the silver coating thickness required for achieving high FOM, the resulting FOM is nearly an order of magnitude greater than the state-of-arts. It is indeed this metaclusters NIMs significant reduction in silver coating thickness that provides the physical basis for the decreased joule heating and thus the realization of ultralow losses. It breaks through the dilemma of whether to use noble metals in engineering visible light metaatom NIMs. In addition, spherically symmetric cluster units directly solve the anisotropy problem of metaatom structure.

### 2.2 Preparation and characterization of meta-cluster particles

**Figure 2:**
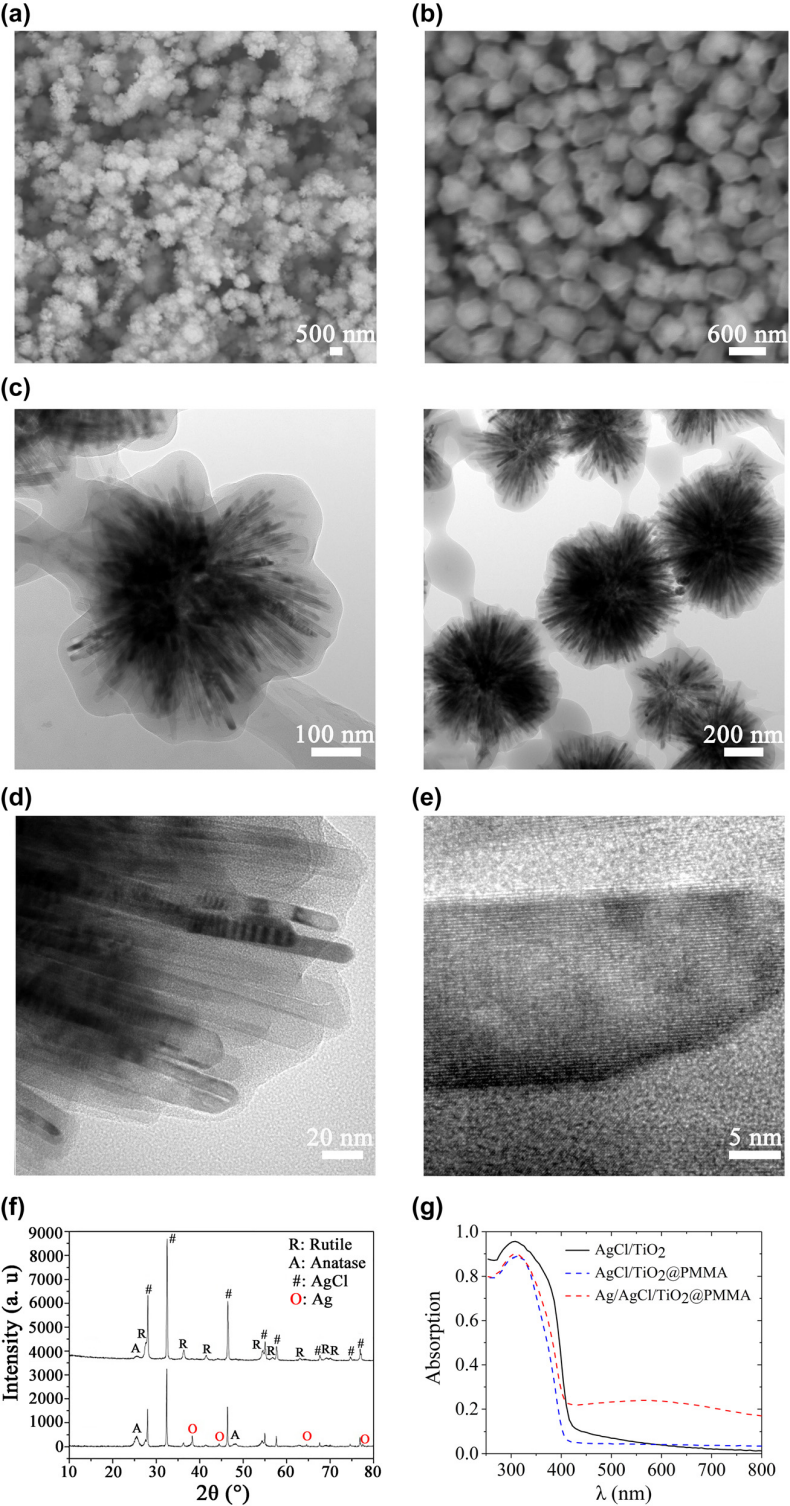
Morphology and characterization of the Ag/AgCl/TiO_2_@PMMA particles. SEM images of (a) AgCl/TiO_2_ particles, (b) Ag/AgCl/TiO_2_@PMMA particles. (c) and (d) TEM images of Ag/AgCl/TiO_2_@PMMA particles: (c) Field of view of green-light (left) and red-light (right) particles; (d) regional view of a composite particle, which is made of a ball-thorn-shaped inorganic kernel and a thin transparent organic PMMA shell. (e) A high magnification TEM image of a protruding nanorod, color variation indicates different chemical compositions. (f) XRD patterns of the AgCl/TiO_2_@PMMA (top) and Ag/AgCl/TiO_2_@PMMA (bottom) particles. After the particles being photoreduction, in addition to the peaks associated with TiO_2_ and AgCl crystals, local maximums of varying intensities (red hollow circles) at 38.3°, 44.4°, 64.6°, and 77.5° – corresponding to the respective (111), (200), (220), and (311) crystal faces of Ag – appeared in the bottom spectral line. (g) UV-VIS-NIR absorption spectra of AgCl/TiO_2_ (solid black line), AgCl/TiO_2_@PMMA (blue dashed line) and Ag/AgCl/TiO_2_@PMMA particles (red dashed line).

**Figure 3:**
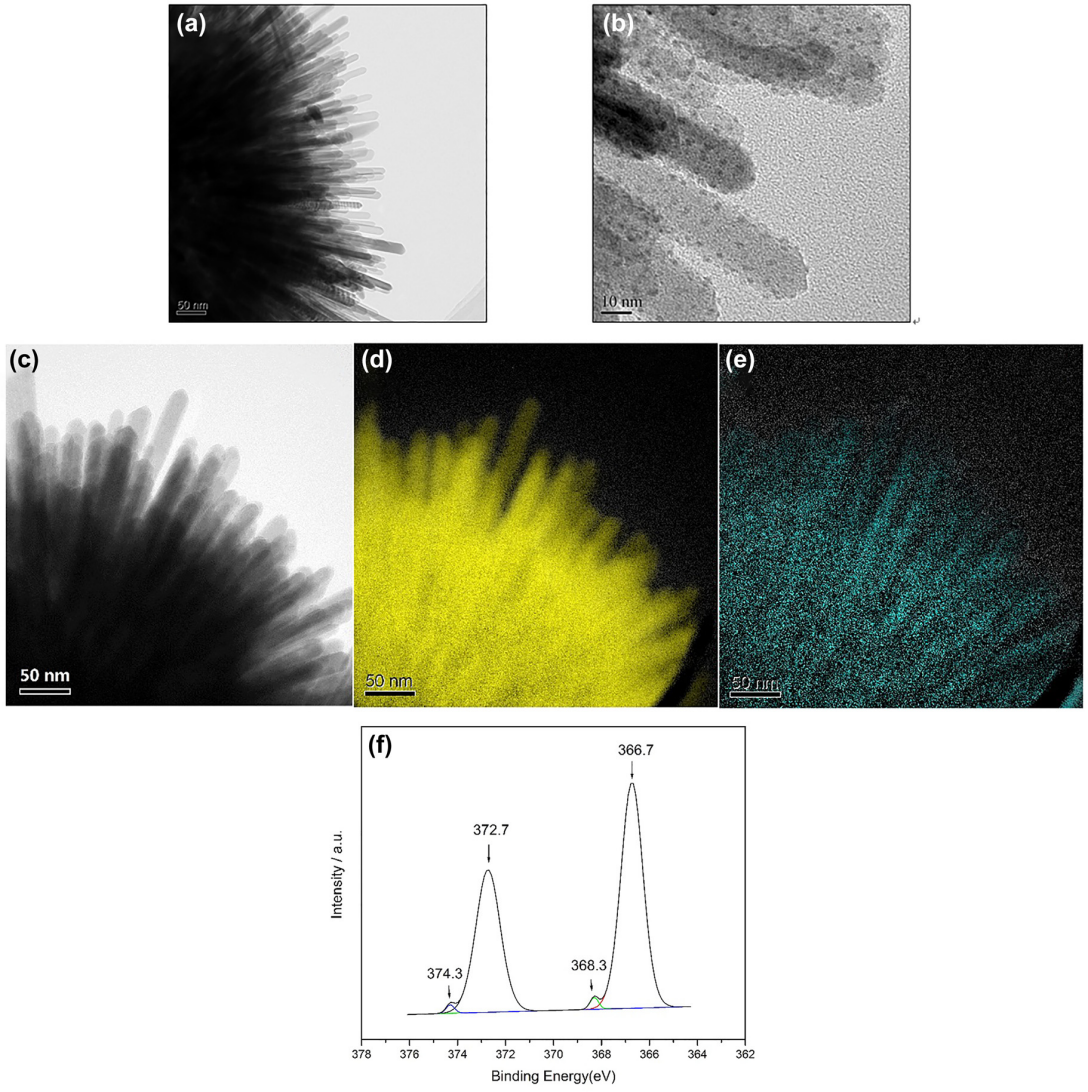
Characterization of the ball-thorn-shaped composite particle. (a) and (b) Local HAADF-STEM images of the Ag/AgCl/TiO_2_ particles, silver appears as surface dispersion distribution on the ball-thorn surface. (c) TEM image of Ag/AgCl/TiO_2_ particle; (d) and (e) the corresponding elemental mapping of Ti and Ag, respectively. (f) XPS analysis of Ag 3d in Ag/AgCl/TiO_2_@PMMA.

The Ag/AgCl/TiO_2_@PMMA metacluster particles corresponding to red-light and green-light are prepared using the solvothermal synthesis method (see Supplementary Information and Methods). In order to solve the problem of the coating of nanosilver layer of ball-thorn-shaped clusters, AgCl is firstly formed by mixing a certain amount of AgNO_3_ into TiCl_4_ during the process of preparing the TiO_2_ rods. After a photoreduction method, AgCl further disintegrates into elemental chlorine and metallic silver. The latter precipitates on the outer surface of the ball-thorn-shaped structure to form the discrete silver distribution about 1 nm. The ball-thorn-shaped particle is shown in the scanning electron microscope (SEM) image (Figure 2a). Next, these agglomerated particles are immersed in PMMA and illuminated to form the Ag/AgCl/TiO_2_@PMMA particles (Figure 2b). Figure 2c shows the TEM images of the particles that resonate in the green (left) and red (right) light spectrum, revealing a classic kernel (AgCl/TiO_2_) – shell (PMMA) structure. The TEM images show that the size of the ball-thorn-shaped Ag/AgCl/TiO_2_ particle is approximately 500–700 nm, and the thickness of the PMMA shell is nearly 20–30 nm. A high-magnification view in Figure 2d confirms the presence of PMMA filling between different nanorods. Another high-magnification TEM image of an individual nanorod shows a rather rough outer surface (Figure 2e), and the local high-angle annular dark-field imaging scanning TEM (HAADF-STEM) images of the Ag/AgCl/TiO_2_ particles exhibits a plaque distribution with different colors (Figure 3a and b), which is possibly a result of the precipitation of Ag nanoparticles. X-ray diffraction (XRD) patterns (Figure 2f) suggests that some AgCl crystals have decomposed into elemental Ag and chlorine, and thus the ultimate post-illumination nanostructure should be Ag/AgCl/TiO_2_@PMMA (Figure 3). Ultraviolet–visible–near infrared (UV-VIS-NIR) absorption spectra of the AgCl/TiO_2_ particles, AgCl/TiO_2_@PMMA particles with no illumination and illuminated AgCl/TiO_2_@PMMA particles (i.e., Ag/AgCl/TiO_2_@PMMA particles) are plotted in Figure 2g. AgCl/TiO_2_ particles only absorb ultraviolet light (i.e., λ < 410 nm). Similar absorption characteristic at ultraviolet light wavelengths is seen for the AgCl/TiO_2_@PMMA particles without going through any illumination (except for a slower varying and slightly reduced magnitude thanks to the PMMA coating). However, after illumination, noticeable difference in absorption is found in the visible light range for the AgCl/TiO_2_@PMMA particles. These illuminated AgCl/TiO_2_@PMMA particles not only exhibit intrinsic absorption behavior of AgCl/TiO_2_ at ultraviolet light wavelengths, but also achieve a wideband absorption in the visible spectrum. Recall the evidence from previous XRD analysis, the increased absorption appeared in the visible light band is likely due to the local plasmon resonance (LPR) of the precipitated Ag nanoparticles. After photochemical reaction in the experiment, silver appears as surface dispersion distribution on the ball-thorn surface.

To further explore the geometric distribution of Ag atoms in the composite particles, Figure 3c–e illustrate the local TEM image of Ag/AgCl/TiO_2_ and the electron energy loss spectroscopy (EELS) elemental mapping of Ti and Ag, correspondingly. In comparison with the TEM image and the elemental mapping of Ti, Ag is uniformly distributed in the entire particle. X-ray photoelectron spectroscopy (XPS) can provide a direct measure of the chemical state of each atom in the compounds. Therefore, the XPS spectra are measured to study the valence state and bonding situation of the Ag elements in the sample. The fitted high-resolution XPS spectra of Ag 3d in Ag/AgCl/TiO_2_@PMMA are depicted in Figure 3f. The two prominent peaks located at 366.7 and 372.7 eV can be attributed to Ag^+^. In addition, the two weak doublets assigned to Ag^0^ at 368.3 and 374.3 eV indicate the existence of metallic Ag in the composite particles. It is concluded that the composition of the post-illumination particles is Ag/AgCl/TiO_2_@PMMA, electron microscope analysis shows that discrete silver distribution with a thickness of about 1 nm can be formed (Figures 2e and 3). The Ag layer can generate plasmon resonance when excited by electromagnetic waves, thereby achieving the performance of metamaterials [49, 50]. Note that conventional cell units, such as fish nets and nanowires, are spatially asymmetric, greatly limiting the possibility for self-assembly. On contrary our metaclusters are spherically symmetric, making them perfect candidates for self-assembly.

It can be seen that our metacluster has the same size as the incident wavelength, which is somewhat similar to a photonic crystal. However, metaclusters resonance is generated by thousands of metaatoms in cluster, so no requirement for photonic crystal collective resonance to produce band gap, and there is no limit to the structural rules of stacking during sample preparation and even allow flaws, which makes three-dimensional visible light wavelength of metamaterials can convenient preparation by disorder assemble method.

## 3 Abnormal behavior of 3D negative index metamaterials

### 3.1 Negative refraction in the visible spectrum

**Figure 4:**
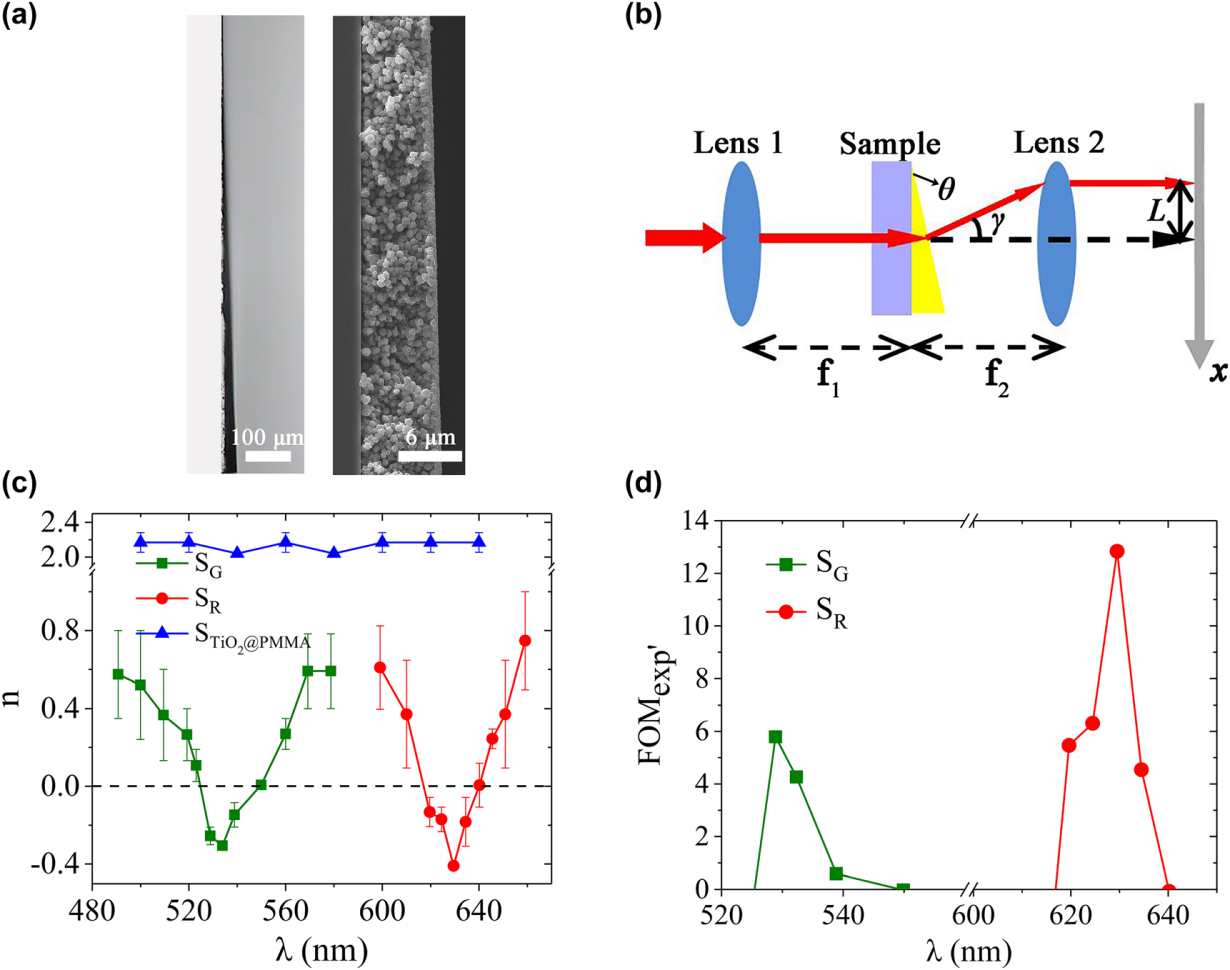
Characterization and measurement of the 3D wedge-shaped samples. (a) Microscopic (left) and SEM (right) images of the wedge-shaped metamaterial sample (side view). (b) Schematic of negative refraction measurement. *θ* represents the wedge angle of the prepared 3D wedge-shaped sample. *γ* refers to the angle between the outgoing beam and the extension line of the incoming beam. *L* represents the offset of the refracted spot in *x*-axis. *f*_1_ = *f*_2_ = 12.7 mm are the focal lengths of lenses 1 and 2, respectively. (c) Measured refractive indices of the green-light sample G, the red-light sample R, and the control sample assembled by TiO_2_@PMMA particles. (d) FOM_exp’_ curves of samples G and R at green-light and red-light wavelengths, respectively.

Red-light and green-light 3D wedge-shaped metamaterial samples were fabricated by assembling the Ag/AgCl/TiO_2_@PMMA particles of different sizes (see Methods and Supplementary Information, Figures S7 and S8, and Table S1). The ∼1° wedge-shaped sample is 5 mm in width, 1 mm in length, and 20 μm in thickness – which is about the height of 30 vertically stacked layers of Ag/AgCl/TiO_2_@PMMA particles (Figure 4a).

Using the method proposed in ref. [16], we designed our own experiment (see Figure 4b and Supplementary Figure S9). Measured refractive indices (see Supplementary Figures S10 and S11, and Tables S2 and S3) of the green-light sample G, the red-light sample R, and the control sample assembled by TiO_2_@PMMA particles are plotted in Figure 4c. The negative refraction for sample R occurs at around 610–640 nm, and the minimum refractive index is −0.41 at 630 nm; the negative refraction for sample G occurs at around 520–550 nm, and the minimum refractive index is about −0.30 at 532 nm. These two measured refractive indices are in a reasonably good agreement with the simulation results (Figure 1e). Measured refractive index STiO_2_@PMMA of the TiO_2_@PMMA sample remains nearly unchanged around a value of 2.2 – close to that of anatase – throughout the visible spectrum, the result of this control sample validates the accuracy of our measurement system. Furthermore, it also demonstrates that the negative refraction observed in the sample R and G indeed originates from the LPR of the topological silver nanoparticles residing on the outer surface of the ball-thorn- particles (consistent with the characterization in Figure 2e–g).

The figure of merit FOM_exp’_ (=−Re (*n*_exp_)/Im(*n*)_sim_ for *n*_exp_<0, and Im(*n*)_sim_ are the imaginary parts of the refractive index obtained by numerical simulation of the meta-clusters) is plotted in Figure 4d. It is worth noting that FOM_exp’_ (≈4.3 at λ = 532 nm and 12.8 at λ = 630 nm) is greater than FOM_sim_ predicted by simulation, which is about 2.2 at λ = 538 nm and about 3.2 at λ = 645 nm where the value of Re(*n*) is the most negative (Figure 1f). This is because we assumed a uniformly distributed Ag layer of 1 nm in thickness when constructing the meta-cluster model in simulation. However, as proven by the TEM images (Figures 2e and 3b), coverage of the Ag layer on the outer surface of the particles is discrete distribution. Less metal presence in the resonance structure likely leads to a reduction in transmission loss caused by volumetric current and plasma resonance. Although replacing Im(*n*) of the experimental value with the simulation value result in a lower FOM in the experiment, yet the comparison between the two is more convenient.

In the experiment, Ag/AgCl/TiO_2_ particles wrapped around PMMA and formed metaclusters. Both red and green light samples were prepared by gravity method using cluster particles. According to SEM photos, the meta-clusters were densely arranged. The refractive index of the sample is determined by the geometrical structure and size of Ag/AgCl/TiO_2_ particles and the properties of PMMA, thus the density of particles have no obvious effect on the wavelength dependent refractive index.

There are direct and indirect methods to test negative refraction in experiments (Table 1). The direct prism test method requires large 3D wedge samples, because of the inevitable cost of high resistive loss, the best result in literature to date is obtained in the infrared band λ = 1.76 μm [16]. For the first time, using a direct method we measured the refractive index of a metamaterial sample at red and green light frequencies. Our ultralow loss, isotropic and three-dimensional large block samples ensure enabled a successful prism measurement. This also further demonstrated that the surface plasma resonance formed by discrete silver layer with the thickness of several stacked atom may instead of the resonant effect of large thick sheets of metal.

**Table 1:** Method and performance comparison of visible metamaterials.

Method	Principle	Structure	Size	Min (*n*’)	Max (FOM)	Refs
Direct measurement	Measurements of the refractive index of these structures were performed by observing the refraction angle of light passing through the prism by Snell’s law [16]	Metacluster composite particles	*t*_Ag_ = 1 nm, *D*_rod_ = 15 nm, *L* = 530/640 nm; *R*_core_ = 165/215 nm	−0.3 (532 nm); −0.41 (630 nm)	4.3 (532 nm); 12.8 (630 nm)	Our work
		Cascaded ‘fishnet’ structures	*t*_Ag_ = 30 nm, period: 860 nm, *t*_MgF2_ = 50 nm	−1.23 (1775 nm)	3.5 (1775 nm)	[16]
	A slit was illuminated by a collimated diode laser beam at different incident angles, and the transmitted light was mapped by scanning a tapered optical fiber at the bottom surface of the metamaterial [40]	Silver nanowires	Diameter: 60 nm Period: 110 nm	−4 (780 nm)		[40]
Indirect measurement	The good agreement between the spectroscopic measurements and the numerical simulation indicates the validity of the numerical model. Therefore, the effective refractive index is calculated using the numerical results and a standard retrieval procedure [51]	‘Fishnet’ structures	*t*_Ag_ = 40 nm, period: 300 nm, *t*_MgF2_ = 17 nm	−0.6 (780 nm)	0.5 (780 nm)	[18]
		‘Fishnet’ structures	*t*_Ag_ = 33 nm, period: 300 nm, *t*_Al2O3_ = 38 nm	−1 (776 nm)	0.7 (772 nm)	[52]
		‘Fishnet’ structures	*t*_Ag_ = 43 nm, period: 220 nm, *t*_Al2O3_ = 45 nm	−0.25 (580 nm)	0.3 (580 nm)	[17]
		‘Fishnet’ structures (incorporate gain media)	*t*_Ag_ = 50 nm, period: 280 nm, *t*_Al2O3_ = 50 nm	−1.26 (738 nm)	10^6^ (738 nm)	[51]
		Multilayered ‘fishnet’ structures	*t*_Ag_ = 35 nm, period: 400 nm, *t*_HSQ_ = 15 nm	−1.3 (752 nm)	3.3 (734 nm)	[53]
		Multilayered ‘fishnet’ structures	*t*_Ag_ = 22 nm, period: 240 nm, *t*_MgF2_ = 15 nm	−0.76 (532 nm)	0.5 (532 nm)	[19]
		‘Fishnet’ structures	*t*_Au_ = 45 nm, period: 200 nm, *t*_air_ = 15 nm	−1.2 (700 nm)	0.5 (700 nm)	[39]
	The refractive index and permeability are retrieved from measured reflection and transmission coefficients using walk-off interferometer [54]	Arrays of silver nanorods	Diameter: 80 nm Length: 650 nm	−0.595 (532 nm) −0.629 (639 nm) −0.899 (690 nm)	2.8 (532 nm) 0.5 (639 nm) 0.8 (690 nm)	[54]

### 3.2 Inverse Doppler effect in visible spectrum

Doppler effect refers to the change of frequency of a wave received by an observer with respect to the wave source when there is a relative movement between each other. It has been widely used in celestial mechanics, medical diagnosis, weather and aviation radar system and many other scientific and engineering fields. Veselago [10] theoretically predicted the existence of inverse Doppler effect in negative refractive index materials. Seddon [11] indirectly realized an inverse Doppler effect at 1–2 GHz using a magnetic nonlinear transmission line for the first time in experiment. Chen et al. [12] observed inverse Doppler effect in the infrared spectrum (λ = 10.6 µm) for the first time by using the two dimensional photonic prism composed of Silicon rods. Recently Shi et al. predicted and demonstrated inverse Doppler effect specific to the Vavilov–Cherenkov cone scenario in a uniform positive refractive index medium [55]. High loss, anisotropy and difficulties in fabricating three-dimensional metamaterials with large areas make experimental observation of inverse Doppler effect in visible spectrum a great challenge that has not been broken through to date.

**Figure 5:**
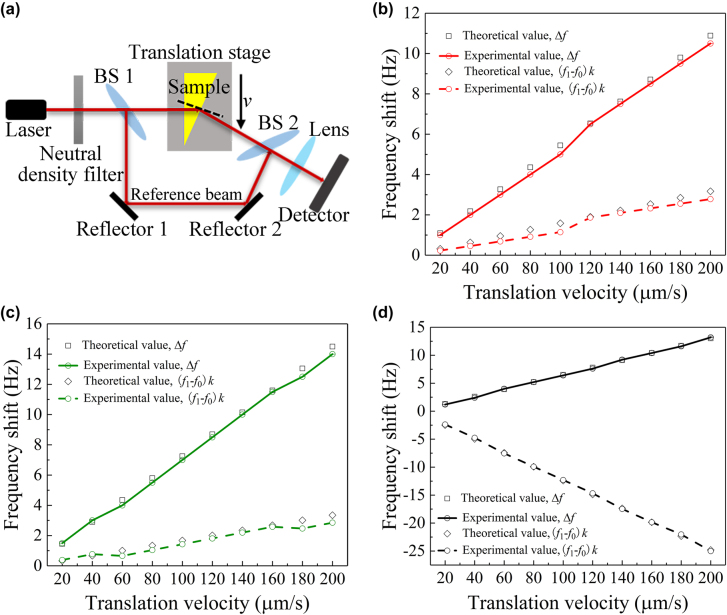
Doppler effect measurement. (a) Schematic diagram of Doppler effect heterodyne detection system. (b)–(d) Beat frequency *∆f* and Doppler frequency shift (*f*_1_–*f*_0_)*k* of Doppler effect observed at different velocities for sample Rc, sample Gc, and TiO_2_@PMMA sample, respectively.

Because the frequency of light is too high to be measured directly, Doppler effect is usually measured by optical heterodyne interferometry [12]. According to the method in ref. [12], we designed a high-precision laser heterodyne detection system based on the refraction of a visible laser beam through the prepared metamaterial wedge-shaped sample (Figure 5a). Uniform change in optical path when light passing through a moving medium results in an observable inverse Doppler effect in visible spectrum (Supplementary Information S4).

The negative refractive index material used for Doppler effect measurement was the ball-thorn shaped Ag/AgCl/TiO_2_@PMMA. To satisfy the width requirement for refractive laser Doppler effect measurement, we prepared two wedge-shaped samples resonating at green light wavelength 525 nm and red light wavelength 632 nm (the bottom size is 5 mm × 2 mm, hence the allowed distance of movement is 2 mm for the laser beam), denoted as sample Gc and sample Rc, respectively. We sent one laser light with a wavelength of 532 nm through the sample Gc (wedge angle *θ* = 1.7, refractive index *n* = −0.3) and another laser light with a wavelength of 632.8 nm through the sample Rc (wedge angle *θ* = 1.4, refractive index *n* = −0.41).

Figure 5b shows the beat frequency of sample Rc at different velocities and the Doppler frequency shift inside the sample. The beat frequency value *∆f* in the graph is obtained by fast Fourier transform of the corresponding signal recorded by the detector. These measured values are in a reasonably good agreement with the theoretical predictions. The experiment was carried out at a sample velocity from 20 to 200 μm/s with a step size of 20 μm/s. We can clearly see that at these speeds, all Doppler shift values $\left({\text{f}}_{1}-{\text{f}}_{0}\right)\text{k}$ for the measured samples are positive, implying the occurrence of inverse Doppler effect. We also observed a similar effect by using sample Gc as shown in Figure 5c.

Figure 5d shows the beat frequency and Doppler frequency shift of the measured TiO_2_@PMMA wedge sample at different velocities. It can be seen that all the Doppler frequency shifts $\left({\text{f}}_{1}-{\text{f}}_{0}\right)\text{k}$ are negative and close to the theoretical values. According to the analysis (Supplementary Information S4), when the source is moving away from the receiver, the Doppler frequency shift value $\left({\text{f}}_{1}-{\text{f}}_{0}\right)\text{k}$ in the sample should be negative, implying red shift in a normal Doppler effect. This result not only demonstrates the feasibility of measuring Doppler effect using a wedge sample but also suggests that our measurement systems is capable of distinguishing inverse Doppler effect from normal Doppler effect. Doppler effect is one of the most basic mechanisms in physics and has wide applications in various fields.

Photonic crystals can have very low loss. However, the band gap of collective resonance of photonic crystals restricts the periodic structure strictly, so the difficulty of preparing photonic crystals is greatly increased, and the best result of inverse Doppler effect experiment found in literature is only 10.6 μm wavelength [12]. The weak interaction between metamaterial units and the behavior of clusters is generated by the local resonance of meta-atoms. Meta-clusters can be arranged in aperiodic structure without mutual restriction effect, which reduces the requirement of integrity and provides the foundation of bottom-up disordered nano-assembly. This ultralow loss, isotropic, 3D large size metamaterial sample prepared using our novel fabrication method confirms the inverse Doppler effect in the visible spectrum for the very first time, which forms a solid foundation for its future application.

## 4 Conclusions

In summary, inspired by the ciliated cell structure, we propose an ultralow loss metaclusters NIMs in the visible spectrum, which is based on meta-atom of the metallic wires [8] and split-rings [9] and the double fishnet of metal–dielectric–metal [16, 18]. The ball-thorn-shaped metaclusters were composed of the dielectric and the discrete super-thin silver layer on its surface, just as internal cytoplasm and surface plasmalemma of the ciliated cell structure. The phenomenon of surface plasmon resonance excited by discrete silver layer with a thickness of several atomic layers produced by the photoreduction method was found, which greatly reduces the generation of loss and breaks through the bottleneck of achieving ultralow loss of optical metamaterials such as noble metals silver and gold. The symmetrical spatial distribution of U-shaped rings and equivalent rods in metacluster provides the isotropy of optical response behavior, and solves the directional problems that always exist in the wires [8] and split-rings [9] units and the double fishing nets [18, 19]. The resonance of 1200 subwavelength atoms in a wavelength scale metacluster provides the physical basis for the external field response of the cluster, the resonance requirements of the whole structure in photonic crystals are not required. Therefore, the difficulty of nano-assemble of these structures is greatly reduced, and a new approach to fabricate visible light metamaterials in large block is provided. These findings offer the striking advances in understanding on light–matter interaction at the nanoscale. Although our demonstration was carried out in the visible range, the design principles should be generalizable to other frequency regimes, such as terahertz and infrared frequencies. Lastly, since propagating waves with large wave vectors are supported in this type of low loss metamaterials, manipulating visible light at subwavelength scale may become possible [14]. This paves the way for a host of emerging technologies such as optical cloaking [7] and plasmonic devices [3], and architected nanolattices have the potential to serve as new optical components and devices [4].

## 5 Methods

### 5.1 Preparation of the meta-cluster particles

*First, Ball-thorn-shaped AgCl/TiO*_*2*_ *particles preparation.* The titanium tetrachloride (TiCl_4_) is added dropwise to deionized water (analytical reagent) under ice bath to prepare a 38.5 wt% solution. The silver nitrate (AgNO_3_, analytical reagent) is dissolved in deionized water to prepare a solution with a concentration of 0.0395 g/mL. The AgNO_3_ solution is added to the tetrabutyl titanate (TBT) and toluene mixture and stirred for 30 min. A certain amount of TiCl_4_ solution is also added and stirred for 1 h. The mixture is transferred to a Teflon-lined autoclave. The reactor is placed in a constant-temperature drying oven (101A-1E) at 150 °C for 24 h. The obtained product is washed several times with absolute ethanol (EtOH, analytical reagent), and then dispersed in ethanol for use or filtered and air-dried to obtain AgCl/TiO_2_ particles. 1.7–2 mL of TiCl_4_ solution is added when preparing the red-light particles, and 1.3–1.5 mL of TiCl_4_ solution is added when preparing the green-light particles.

*Second, Functionalization of AgCl/TiO*_*2*_ *particles.* A certain amount of the prepared AgCl/TiO_2_ particles are added into the EtOH to obtain a 50 mL suspension. The suspension is then transferred into a 100 mL three-necked flask and stirred at 90 rpm for 30 min. 2 mL of polyethylene glycol-400 (PEG-400, analytical reagent) is dissolved in 5 mL of EtOH and slowly dropped in the three-necked flask. After stirring the suspension for 1 h, 1 mL of *γ*-methacryloxy propyltrimethoxy silane (MPS, analytical reagent) is dissolved in 5 mL of EtOH and slowly added into the three-necked flask. Similarly, after stirring the suspension again for 5 h, 1 mL of ammonium hydroxide (25 wt%) is dissolved in 5 mL of EtOH and slowly dropped into the three-necked flask. After being stirred for 10 h, the suspension is centrifuged at a rate of 2200 rpm for 3 min to discard the supernatant. The procedure is repeated 2 to 3 times, the precipitated MPS-functionalized AgCl/TiO_2_ particles are obtained.

*Third, PMMA-coated AgCl/TiO*_*2*_ *particles (AgCl/TiO*_*2*_*@PMMA).* The AgCl/TiO_2_@PMMA composite particles were synthesized by the route that the monomer was adsorbed onto the modified AgCl/TiO_2_ followed by dispersion polymerization. A certain amount of the functionalized AgCl/TiO_2_ particles is transferred to a 250 mL three-necked flask. 2 mL of methyl methacrylate (MMA, analytical reagent) and 10 μL of ethylene glycol dimethacrylate (EGDMA, analytical reagent) are dissolved in 25 mL of EtOH. The mixture is then slowly dropped in the three-necked flask. After stirring the suspension in the three-necked flask at 90 rpm for 1 h, 0.2 g of polyvinyl pyrrolidone (PVP, analytical reagent) is dissolved in 80 mL of deionized water and added to the three-necked flask using a funnel. The suspension is continuously stirred for 1 h, and the three-necked flask is transferred to a thermostat water bath (80 °C) and condensed with nitrogen. Subsequently, 0.06 g of kalium persulfate (KPS, analytical reagent) is dissolved in 6 mL of deionized water. Under constant stirring, 6 mL of KPS solution is added to the three-necked flask in three portions: 2 mL is dropped every 2 h. After the last addition of the KPS solution, the suspension is stirred for 6 h to complete the coating of AgCl/TiO_2_ and obtain a suspension of AgCl/TiO_2_@PMMA particles. The resulting suspension is centrifuged at 3000 rpm for 5 min to discard the supernatant. The remaining precipitate is then washed with the deionized water and centrifuged for several times. The final precipitate is washed with a small amount of deionized water before transferring to a 10 mL vial for storage.

*Finally, Ag/AgCl/TiO*_*2*_*@PMMA composite particles.* The quartz glass is hydrophilically treated. The clean quartz glass (1 cm × 2 cm) is sonicated in alcohol for 30 min, washed with deionized water, and then boiled for 1 h in a mixture of 30% hydrogen peroxide (H_2_O_2_, analytical reagent) and deionized water (7:3 by volume). The suspension of the AgCl/TiO_2_@PMMA particles is spin-coated onto a hydrophilically treated glass substrate using a spin coater. Finally, the glass substrate coated with AgCl/TiO_2_@PMMA particles is placed a photoreduction process under an incandescent lamp (or a xenon lamp, λ > 420 nm) for 10 h. Part of AgCl in the particles is decomposed into Ag elementary substance, which is precipitated on the surface of the particles to obtain Ag/AgCl/TiO_2_@PMMA particles.

### 5.2 Characterization

The morphology was observed by scanning electron microscopy (SEM, JSM-6700) and transmission electron microscopy (TEM, JEOL-3010). The crystal structure was characterized by the powder X-ray diffraction (XRD, Philips X’Pert Pro) with CuK_α_ irradiation (40 kV/35 mA) and step size of 0.033° in the 2*θ* range of 10°–80°. Absorbance spectra were measured using UV-VIS-NIR spectrophotometer (HITACHI U-4100).

### 5.3 Preparation of 3D wedge-shaped samples

Gravity self-assembly device (Figure S7) is set as a platform to prepare the wedge-shaped sample. The lifting slab of the experiment platform is adjusted to be horizontal. The 5 mm × 10 mm glass strip is horizontally positioned in the glass substrate, whereas another hydrophilically treated glass strip (20 mm × 40 mm) is vertically placed on the glass strip (5 mm × 10 mm) and pressed down with a proper force to ensure that the suspension will not leak during the painting. Nearly 3.5 μL of the suspension is collected using a pipette and evenly painted from one end to the other along the corner between the two orthogonal glass strips. Under the action of hydrophilicity and gravity, a wedge-shaped suspension is formed. After the water in the wedge-shaped suspension evaporates at room temperature, the horizontal glass strip containing the wedge-shaped sample with Ag/AgCl/TiO_2_@PMMA particles is taken down.

### 5.4 Measurement of the refractive index

The diagram of the experimental setup is displayed in Supplementary Figure S9. The refractive index of the wedge-shaped sample at different incident wavelengths is obtained in accordance with the following formula by changing the incident wavelength and repeating the measurement:

(1) $$n=\text{sin}\left\{\left[\text{arctan}\left(0.625L/f_{2}\right)+\theta \right]\right\}/\text{sin}\left(\theta \right)\text{,}$$

where *L* is the displacement of the refracted spot, *f*_2_ is the distance from lens 2 to the sample, that is, the focal length of lens 2, and *θ* is the wedge angle of sample.

### 5.5 Numerical simulations

The boundary conditions of the model are set as perfect electric conductor (PEC) in the *x* direction, perfect magnetic conductor (PMC) in the *y* direction, and Open in the *z* direction which is also the direction of the incident light beam. This metacluster unit cell model is then solved using the time domain solver in CST Microwave Studio. Based on the Mie theory, the effective parameters are retrieved from the simulation results. The retrieve method is introduced in the Supporting Information S1.

## Supplementary Material

Supplementary Material
