# Supplementary material for: Ultralow loss visible light metamaterials assembled by metaclusters
Source: Nanophotonics. 2022 May 13;11(12):2953–66. doi: 10.1515/nanoph-2022-0171 (PMC11501988; doi:10.1515/nanoph-2022-0171)
Supplement: Supplementary file 1 — Supplementary Material [file j_nanoph-2022-0171_suppl.docx]

Supplementary Information

**Ultralow Loss Visible Light Metamaterials Assembled by Meta-clusters**

Jing Zhao^1^*, Huan Chen^2^, Kun Song^2^, Liqin Xiang^2^, Qian Zhao^3^, Chaohong Shang^2^, Xiaonong Wang^2^, Zhijie Shen^2^, Xianfeng Wu^2^, Yajie Hu^2^ and Xiaopeng Zhao^2^*

^1^Medtronic plc, Boulder, CO 80301, USA.

^2^Smart Materials Laboratory, Department of Applied Physics, Northwestern Polytechnical University, Xi’an 710129 P. R. China.

^3^State Key Lab Tribology, Dept Mech Engn, Tsinghua University, Beijing 100084, P. R. China.

Jing Zhao, Huan Chen and Kun Song contributed equally to this work.

*Corresponding author. E-mail: xpzhao@nwpu.edu.cn; zhaojing1120@gmail.com.

**This Supplementary Information document includes:**

S1. Bionic model and simulation

S2. Preparation and characterization of the meta-cluster particles

S3. Preparation of the 3D wedge-shaped samples and measurement of the refractive index

S4. Measurement of the Doppler effect

**S1. Bionic model and simulation**

1. **Structure and function of the cilia on biological cells**

Biological research has shown that swayable and slender protuberances projecting from the cell body are present on the surface of prokaryotic and eukaryotic cells; such protuberances are called cilia. Under the optical microscope, one cell can have several hundreds of cilia with a 5–10 μm length, a 0.15–0.3 μm diameter, and a dense particle at the root (i.e., basal body). A cilium consists of three main parts, namely, the microtubule-based cytoskeleton called the axoneme, the plasma membrane surrounding the axoneme, and some cytoplasm (Figure1a). The axoneme is a bundle of microtubes with a diameter of approximately 220–240 Å from the basal body to the top of the cilium. At the bottom of the basal body, axonemes are concentrated into a conical bundle that penetrates into the cytoplasm. The cilia can rhythmically oscillate in a certain direction, thus pushing the secretions and particulate matter that adhere to the epithelial cells in a certain direction. For example, most of the cavity surface of the respiratory tract is composed of ciliated epithelium, and the inhaled dirt and bacteria can be discharged given the directional oscillation of the cilia.

1. **Influence of structural and material property parameters on the optical response behavior of the meta-cluster model**

The final numerical simulation results of the red model and green model. A peak in transmission coefficient magnitude accompanied by an abrupt phase shift in the vicinity of 640 nm can be clearly seen from the simulated transmission coefficient and reflection coefficient curves (Figure 1c) when a light wave is incident on the meta-cluster structure with *l* = 640 nm, *r* = 215 nm and *P* = 670 nm (t_Ag_= 1nm for unspecified cases). This indicates the meta-cluster structure resonates within the wavelength range of the red-light. Based on the Mie scattering theory, the effective parameters (Figure 1d) are numerically retrieved from the simulated transmission and reflection coefficients, resulting in simultaneously negative permeability, permittivity and refractive index at around 640 nm, which proves that the material composed of this structure is a metamaterial. At λ = 645 nm, the value of Re(n) reaches a minimum of −0.45. To achieve a similar effect in the green-light band, we reduced the diameter *l* of the meta-cluster to 530 nm and set *r* = 165 nm, *P* = 560 nm. As expected, the simulated transmission coefficient and reflection coefficient curves (Figure S1e) indeed reveals a Mie resonance at the green-light wavelengths. Similarly, derived from the Mie scattering theory, the effective permeability, permittivity and refractive index of the meta-cluster structure are simultaneously negative at near 530 nm (Figure S1f). At λ = 538 nm, the value of Re(n) reaches a minimum of −0.47.


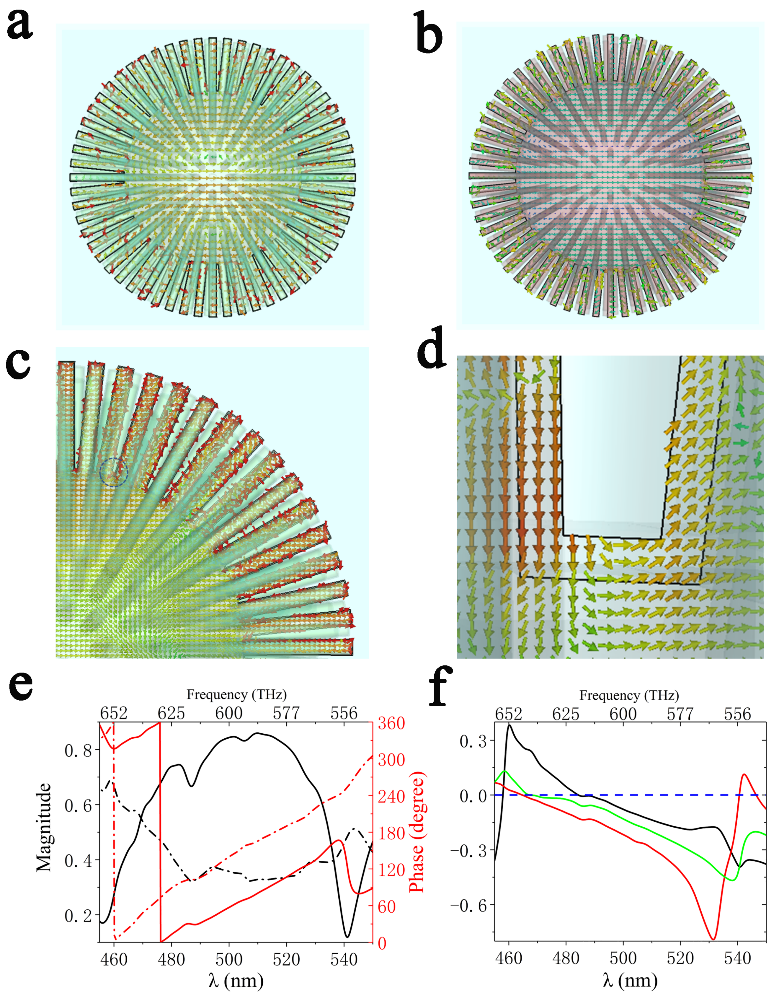


**Figure S1.** **Numerical simulation of the meta-cluster structure. a, b**, The profile current distribution perpendicular to the external magnetic field of green and red meta-clusters respectively. **c**, Green meta-clusters 1/4 profile current distribution. **d**, Local enlarged view (dashed line frame in **c**), showing ring current. **e.** Transmission (solid line) and reflection (dot-dash line) coefficient for the green-light meta-clusters with *l* = 530 nm, *r* = 165 nm, and *P* = 560 nm. **f.** The effective parameters for the green-light meta-clusters retrieved from the coefficients in **e**.

1. **Red-light meta-cluster structure model**

**A.** Air medium

A meta-cluster structure model is designed, as shown in Figure S2a. The diameter *l* of the entire structure is 640 nm, and the radius *r* of the spherical kernel is 200 nm. The diameter D of the rod is 15 nm, and the number of the rods on the spherical kernel is 600. In addition, the lattice constant *P* is 670 nm. The medium around the meta-cluster structure is air. The calculated transmission and reflection coefficient curves when the permittivity of TiO_2_ is set to 5, 5.2, and 5.5 are presented in Figure S2b, b, and d, respectively. In these figures, the solid lines represent the transmission coefficient, and the dotted lines represent the reflection coefficient. The curves suggest that the meta-cluster structure resonates in the red band, and the transmission coefficient has obvious transmission peak and abrupt phase shift. The resonant wavelength is red shifted with the increase in the permittivity of TiO_2_. The effective parameters of the designed meta-cluster structure are retrieved using Mie theory [41,48]:

$Zeta=\sqrt{\frac{(1+\mathrm{r})^{2}-t^{2}}{(1-\mathrm{r}^{2})-t^{2}}}$, (1)

$k_{d}=\frac{1}{d}\arccos\left[\frac{(1-\mathrm{r}^{2})+\mathrm{t}^{2}}{2t}\right]$, (2)

$\mu=\frac{k_{d}\times Zeta}{k}$, (3)

$\varepsilon=\frac{(k_{d}/k)^{2}}{\mu}$, (4)

$n=Zeta\times\varepsilon$, (5)

where r is the reflection coefficient, t represents the transmission coefficient, d is the thickness of the sample, and k = 2π*f*/c (*f* is the frequency of the incident light and c represents the light speed in vacuum space). The retrieved results are shown in Figure S2e, f, and g. The permeability, permittivity, and refractive index of the meta-cluster structure are negative at approximately 640 nm. Moreover, the position of the refractive index valley becomes red shifted with the increase in the permittivity of TiO_2_.


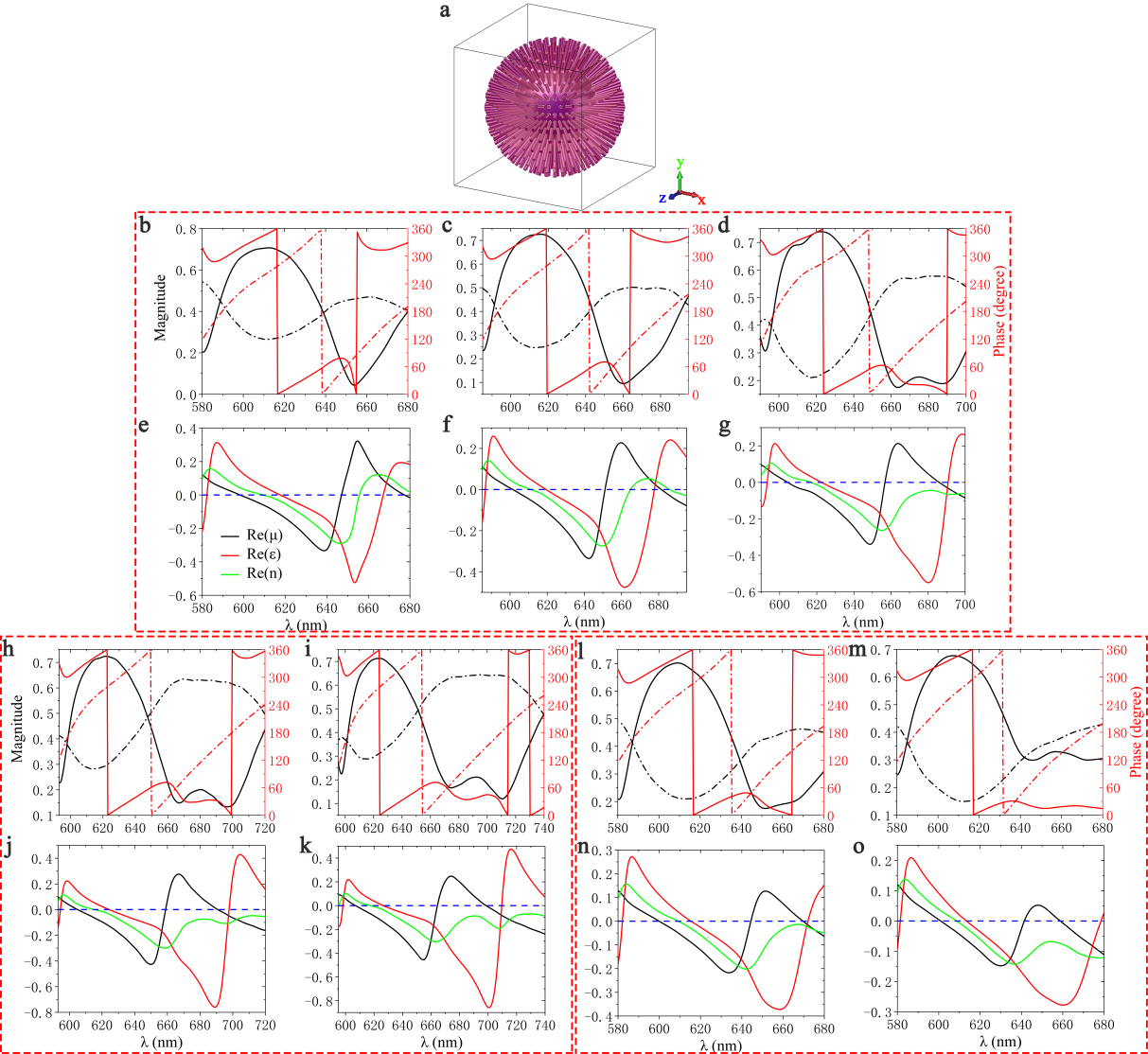


**Figure S2. Effect of structural and material property parameters on the response behavior of the red-light meta-cluster in air medium. a,** The meta-cluster model in air medium. **b**, **c**, and **d** Transmission and reflection coefficient curves when the permittivity of TiO_2_ is set to 5, 5.2, and 5.5, correspondingly. **e**, **f**, and **g** Permeability, permittivity, and refractive index curves retrieved based on the results in **b**, **c**, and **d**, respectively. **h**, **i** Transmission and reflection coefficient curves of the meta-clusters with *r* = 210 and 215 nm, respectively. **j**, **k** Permeability, permittivity, and refractive index curves retrieved through the results presented in **h** and **i**, respectively. **l**, **m** Transmission and reflection coefficient curves of the meta-clusters when the number of rods is 700 and 800, respectively. **n**, **o** Permeability, permittivity, and refractive index curves retrieved through the results presented in **l** and **m**, respectively.

The permittivity of TiO_2_ is set to 5.2. The number of rods is 600, *l* = 640 nm, *P* = 670 nm. The corresponding transmission and reflection coefficient curves are calculated with *r* = 210 and 215 nm, as shown in Figure S2h and i. The resonant wavelength undergoes a small redshift with the increase in *r*. The permeability, permittivity, and refractive index curves, which are retrieved using Mie theory, are illustrated in Figure S2j and k. Through the comparison of Figure S2f with Figure S2j and k, the position of the refractive index valley is redshifted, and the absolute value of the refractive index valley slightly increases with *r*.

The permittivity of TiO_2_ is set to 5, *r* = 200 nm, *l* = 640 nm, and *P* = 670 nm. The number of rods on the spherical kernel is set to 700 and 800. The calculated transmission and reflection coefficient curves are presented in Figure S2l and m. As the number of rods increases, the valley of the transmission coefficient amplitude increases; the abrupt phase shift of the transmission coefficient decreases, and the position of this shift exhibits a small blue shift. The permeability, permittivity, and refractive index curves are obtained using these findings (Figure S2n and o). With the increase in the number of rods, the absolute values of permeability, permittivity, and refractive index decrease; the position of the refractive index valley demonstrates a small blue shift.

**B.** PMMA medium

We then set the medium around the meta-cluster structure to PMMA (Figure S3a). Here, *r* = 200 nm, *l* = 640 nm, *P* = 670 nm, and the number of rods is 600. In addition, ε_PMMA_ is set to 2.4, and the permittivity of TiO_2_ is changed. The respective simulation results when the permittivity of TiO_2_ is set to 5, 5.2, and 5.5 are demonstrated in Figure S3, b–d. The structure resonates in the red band, and a large abrupt phase shift occurs. With the increase in the permittivity of TiO_2_, the resonant wavelength is redshifted. The results retrieved using Mie theory are displayed in Figure S3e–g. These results indicate that the permeability, permittivity, and refractive indexes of the meta-cluster structures are negative at approximately 640 nm. With the increase in the permittivity of TiO_2_, the absolute value of the permittivity valley significantly increases, and the position of the valley is redshifted. Moreover, the absolute value of the refractive index valley slightly increases and the position of the valley is likewise red shifted.


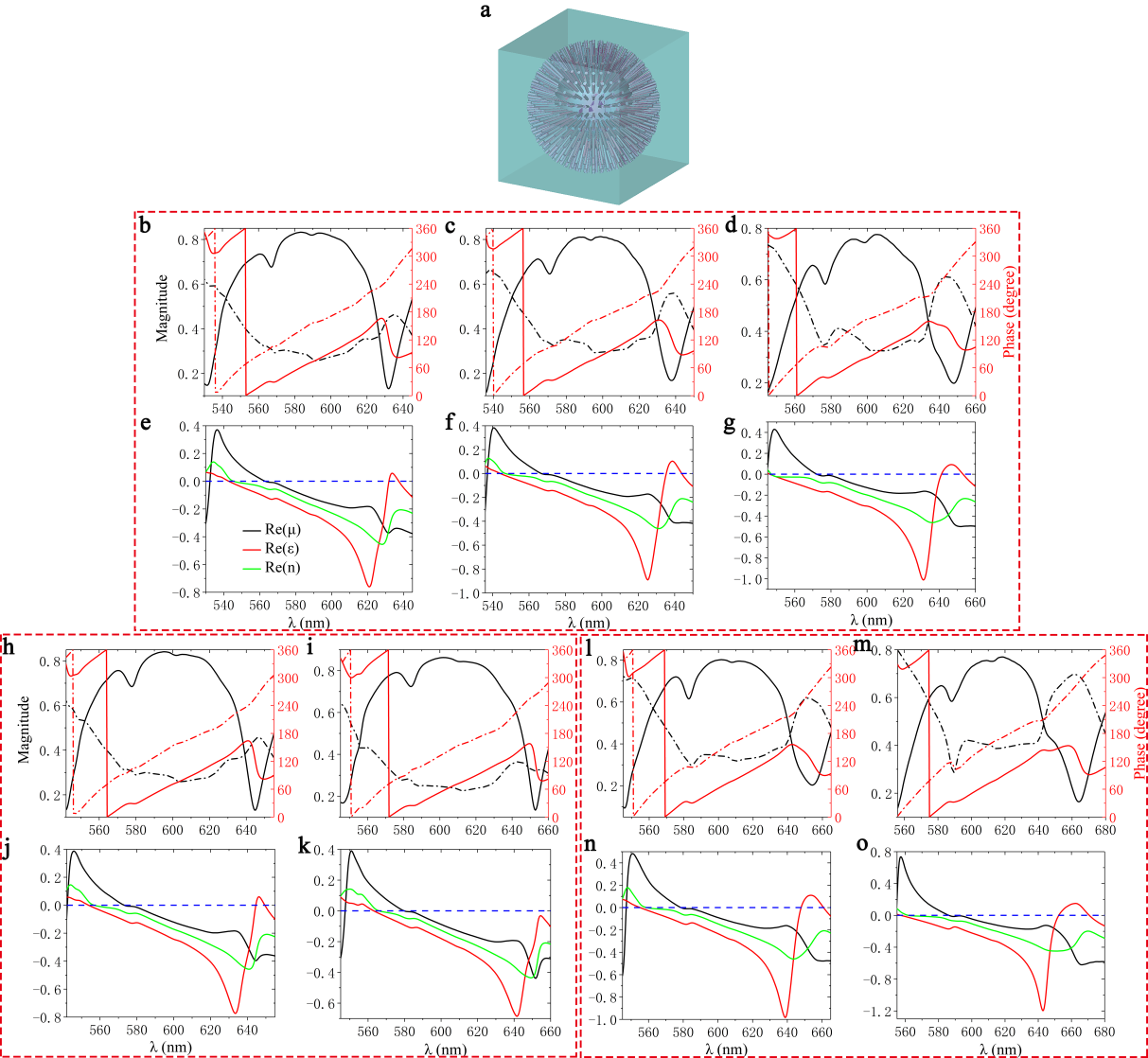


**Figure S3.** **Effect of structural and material property parameters on the response behavior of the red-light meta-cluster in PMMA medium.** **a**, The meta-cluster model in PMMA medium. **b**, **c**, **d**, Transmission and reflection coefficient curves of the meta-clusters when the permittivity of TiO_2_ is set to 5, 5.2, and 5.5, respectively. **e**, **f**, **g**, Permeability, permittivity, and refractive index curves retrieved based on the results in **b**, **c**, and **d**, respectively. **h**, **i**, Transmission and reflection coefficient curves of the meta-clusters when ε_PMMA_ is set to 2.5 and 2.6, respectively. **j**, **k**, Permeability, permittivity, and refractive index curves retrieved from the results presented in **h** and **i**, correspondingly. **l**, **m**, Transmission and reflection coefficient curves of the meta-clusters when *r* is 210 and 220 nm, respectively. **n**, **o**, Permeability, permittivity, and refractive index curves retrieved from the results presented in **l** and **m**, correspondingly.

The permittivity of TiO_2_ is set to 5.2. The geometric parameters of the meta-cluster structure are the same as those of the structure presented in Figure S3a, and ε_PMMA_ is then varied. The transmission and reflection coefficient curves obtained through simulation when ε_PMMA_ = 2.5 and 2.6 are shown in Figure S3h and i, correspondingly. Both figures show that the resonance peak of the meta-cluster structure and the position of the abrupt phase shift are redshifted with the increase in ε_PMMA_. Mie theory is used again to retrieve the effective parameters from the simulation results (Figure S3j and k). With the increase in ε_PMMA_, the absolute values of the permittivity and the refractive index valleys slightly decrease, and the positions of the valleys are redshifted.

The permittivity of TiO_2_ is set to 5.2, ε_PMMA_ = 2.5, *l* = 640 nm, *P* = 670 nm, the number of rods is 600. The corresponding transmission and reflection coefficient curves are obtained through simulation with *r* = 210 and 220 nm, as demonstrated in Figure S3l and m. These results suggest that the resonant wavelength is redshifted with the increase in *r*. These curves are then utilized to retrieve the permeability, permittivity, and refractive index curves (Figure S3n and o). With the increase in *r*, the position of the refractive index valley is redshifted, and the absolute value of the permittivity valley evidently increases.

1. **Green-light meta-cluster structure model**

In this model, *l* = 530 nm, *r* = 165 nm, P = 560 nm, and the number of rods is 600. In addition, the permittivity of TiO_2_ is set to 5.2, and ε_PMMA_ = 2.4. The obtained transmission and reflection coefficient curves are illustrated in Figure S4a. The resonant wavelength is redshifted with the increase in ε_PMMA_. Figure S4b presents the effective parameters retrieved using Mie theory, thereby indicating that the permeability, permittivity, and refractive index of the meta-cluster structure are negative at nearly 530 nm, and the refractive index is nearly the same as that measured in the experiment. Moreover, with the increase in ε_PMMA_, the positions of the permittivity and refractive index valleys demonstrate a significant red shift, and the absolute values of the valleys slightly decrease.


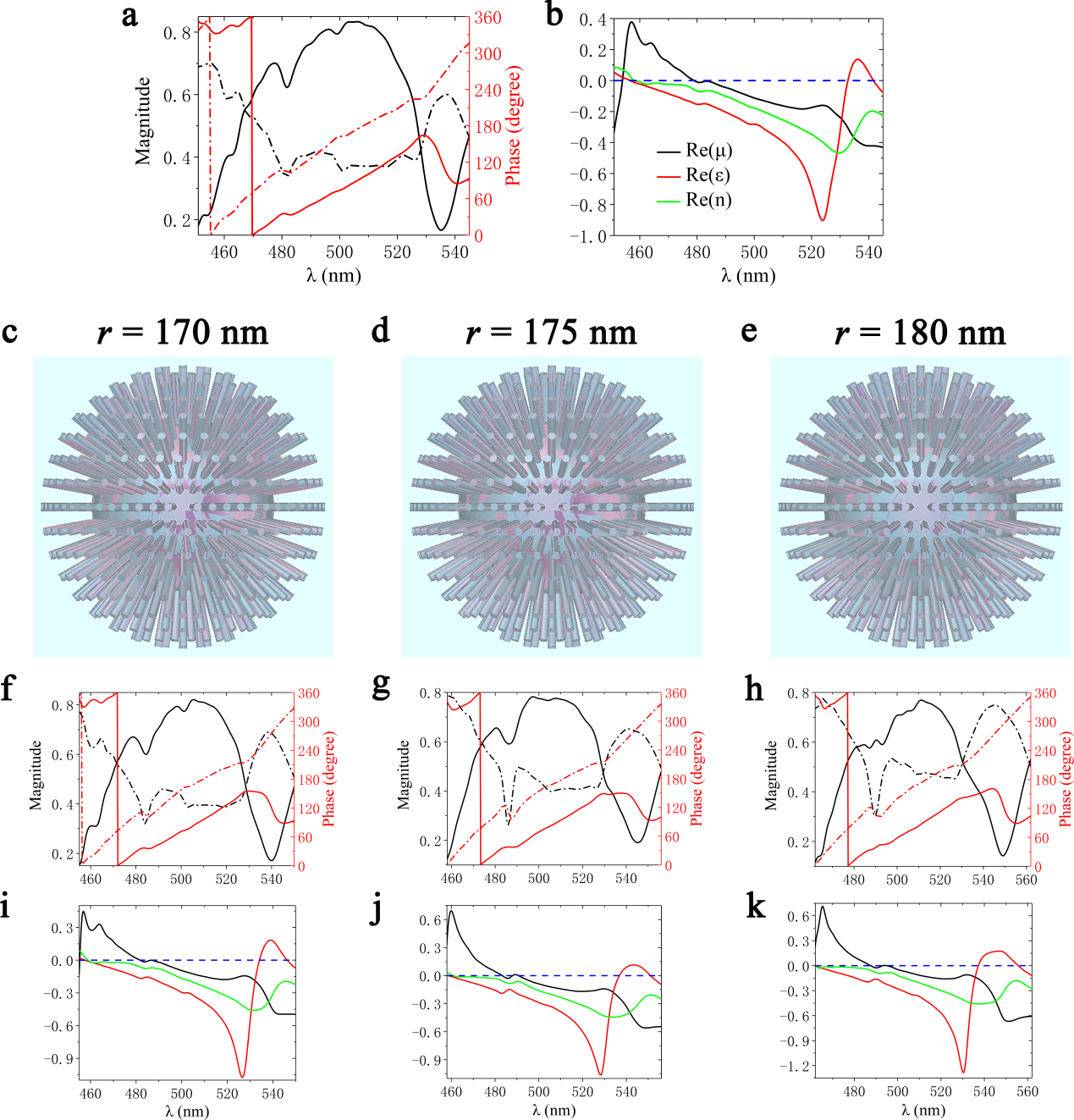


**Figure S4. Effect of structural and material property parameters on the response behavior of the green-light meta-cluster in PMMA medium.** **a**, Transmission and reflection coefficient curves, **b**, permeability, permittivity, and refractive index curves of the meta-clusters when ε_PMMA_ = 2.4. **c**, **d**, **e**, Front views of the meta-cluster structures when *r* = 170, 175, 180 nm, respectively. **f**, **g**, **h**, Transmission and reflection coefficient curves of the meta-clusters in **c**, **d**, and **e**, correspondingly. **i**, **j**, **k**, Permeability, permittivity, and refractive index curves of the meta-clusters in **c**, **d**, and **e**, respectively.

And then *r* is changed, as shown in Figure S4c, d, and e, corresponding to *r* = 170, 175, and 180 nm, respectively. The corresponding transmission and reflection coefficient curves of the meta-cluster structures are obtained through simulation (Figure S4f–h). These curves indicate that the resonant wavelength is red shifted with the increase in *r*. The retrieved permeability, permittivity, and refractive index curves of the meta-cluster structures are exhibited in Figure S4i–k. These curves indicate that, with the increase in *r*, the positions of the permittivity and refractive index valleys undergo a small red shift, and the absolute value of the permittivity valley significantly increases.

1. **Influence of Ag layer thickness on the FOM of the fishnet structure metamaterial**


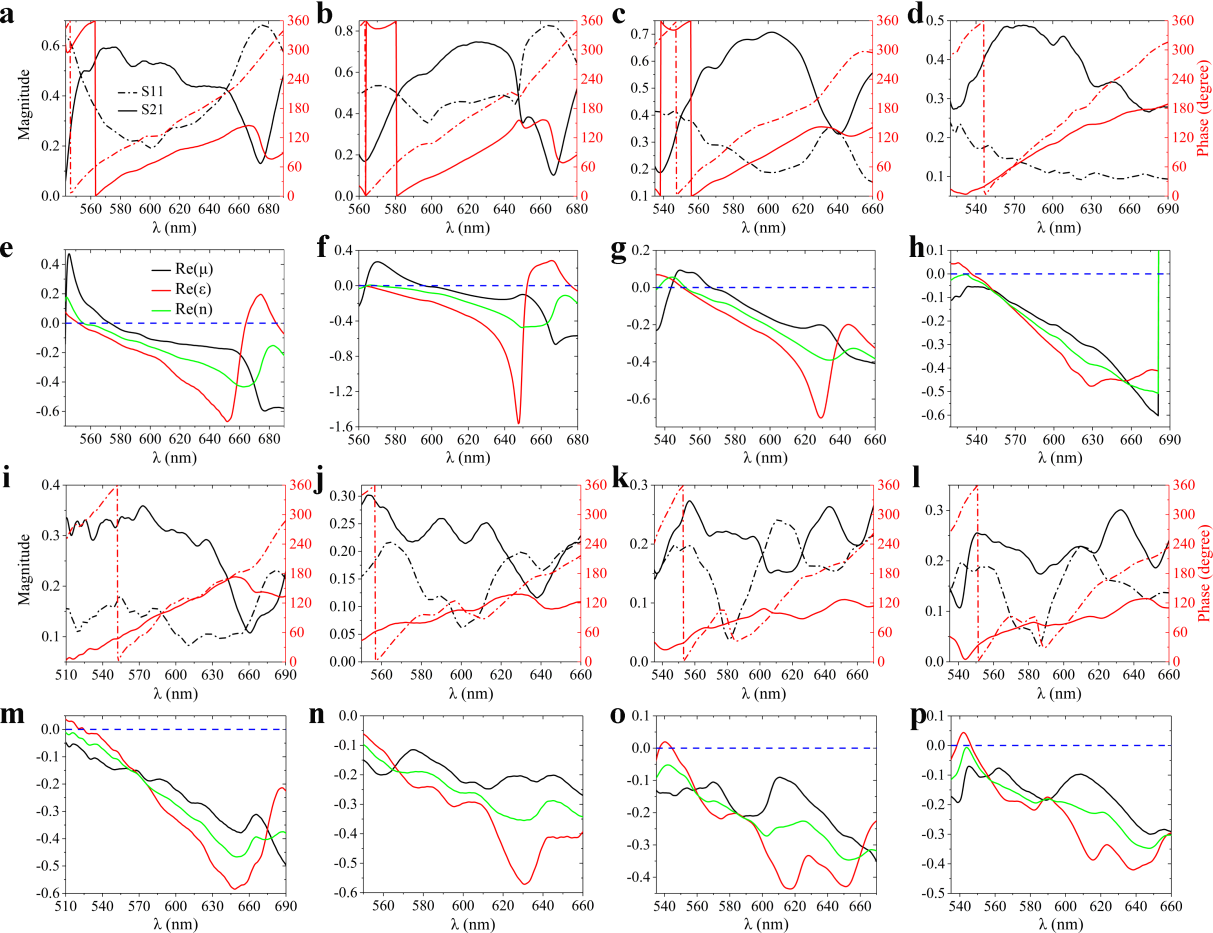


**Figure S5.** **Effect of Ag** **layer thickness t_Ag_ on the response behavior of the red-light meta-cluster in PMMA medium.** **a**, **b**, **c**, **d**, Transmission and reflection coefficient curves of the meta-clusters with t_Ag_ = 0.5, 0.7, 2, and 3 nm respectively. **e**, **f**, **g**, **h**, Permeability, permittivity, and refractive index curves of the meta-clusters retrieved from **a**, **b**, **c**, and **d**, respectively. **i**, **j**, **k**, **l**, Transmission and reflection coefficient curves of the meta-clusters with t_Ag_ = 4, 5, 6, and 7 nm respectively. **m**, **n**, **o**, **p**, Permeability, permittivity, and refractive index curves of the meta-clusters retrieved from **i**, **j**, **k**, and **l**, respectively.

In the text, we have researched the effect of the Ag layer thickness on the FOM of the meta-cluster structure (Figure 1e and Figure S5). As a comparison, here the effect of Ag layer thickness on the FOM of the following two fishnet structures is investigated. Based on the fishnet structure model proposed by Xiao *et al.*, [17] the fishnet structure unit shown in Figure S6a is designed. The structure unit is a sandwich structure, that is, Ag-Al_2_O_3_-Ag structure. The thickness of Al_2_O_3_ is 45 nm, the width of the nanostrip is 140 nm, and the period of the unit is 220 nm. The thickness of one of two same Ag layers is t_Ag_. The fishnet structure metamaterials with different Ag layer thickness are simulated in the CST microwave studio by the frequency domain solver. The boundaries in the x and y directions are set as Perfect Electric Conductor (PEC) and Perfect Magnetic Conductor (PMC) boundaries, and the boundaries in the z direction are set as open boundaries. The refractive index of Al_2_O_3_ with the loss factor of 0.001 is set to 1.76, and the permittivity of Ag is set to the actual Drude value [47]. When the thickness of Ag layer is 2, 3, 5, 10, 13, 14, 17, 20, 30, 35, 40, and 43 nm respectively, the FOM of the corresponding structure in the red or near-infrared band is obtained by simulation (the effective parameters are obtained through effective medium theory, and Figure S6b presents the retrieved effective parameters of the fishnet metamaterial with t_Ag_=17 nm), as shown in Figure S6c. It is found that with the increase of Ag layer thickness, the maximum FOM (FOM_max_, indicating by the black dotted line) of fishnet structure increases rapidly at first, and then decreases slowly after reaching the peak (about 1.76) at the thickness of approximately 17 nm. The red dotted line represents the FOM at the position where the refractive index of the fishnet structure is the most negative, and its changing trend is consistent with the black dotted line. In addition, we also simulated and calculated the effective parameters of the fishnet structure when the thickness of the Ag layer was 0.5 and 1 nm respectively; the results showed that the refractive index was positive in the entire visible and near-infrared bands.


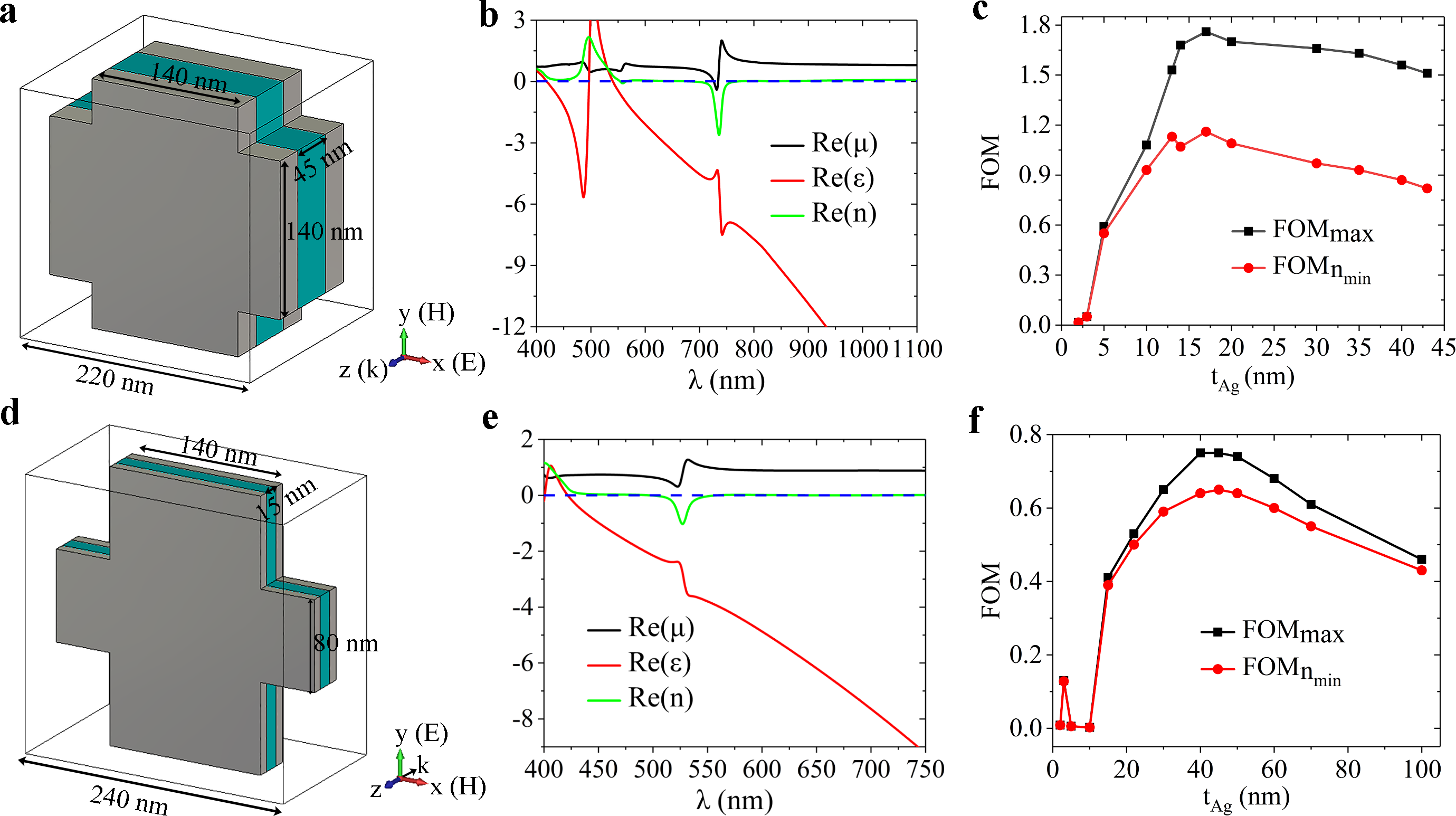


**Figure S6. Numerical simulation of two fishnet structure metamaterials. a**, **b**, and **c** Fishnet structure model (Ag-Al_2_O_3_-Ag structure, and the geometrical parameters refer to the published work [17]), retrieved effective parameters at t_Ag_ =17 nm, and FOMs at different Ag layer thickness, respectively. **d**, **e**, and **f** Fishnet structure model (Ag-MgF_2_-Ag structure, and the geometrical parameters refer to the published work [19]), retrieved effective parameters at t_Ag_ =45 nm, and FOMs at different Ag layer thickness, respectively. The black dotted lines represent the maximum FOM (FOM_max_) of fishnet structure, and the red dotted lines represent the FOM at the position where the refractive index of the fishnet structure is the most negative.

In order to study the influence of the thickness of the Ag layer on the FOM when the medium layer in the fishnet structure is relatively thin, we design the fishnet structure unit shown in Figure S6d based on the fishnet structure model proposed by Liang *et al.* [19]. Similarly, the structure is also a sandwich structure, namely Ag-MgF_2_-Ag structure. The thickness of MgF_2_ is 15 nm. In the fishnet structure, the width of the nanostrip along the x direction is 140 nm, the width of the nanostrip along the y direction is 80 nm, and the period is 240 nm. The simulation is also conducted in the CST microwave studio by the frequency domain solver. The boundaries in the x and y directions are set as PMC and PEC boundaries respectively, and the boundaries in the z direction are set as the open boundaries. The light is incident perpendicular to the fishnet structure along the -z direction. The refractive index of MgF_2_ is set to 1.38, and the permittivity of Ag is set to the actual Drude value [47]. When the thickness of Ag layer is 2, 3, 5, 10, 15, 22, 30, 40, 45, 50, 60, 70, and 100 nm respectively, the FOM of the corresponding structure in the red or near-infrared band is obtained by simulation (the effective parameters are also obtained through effective medium theory, and Figure S6e presents the retrieved effective parameters of the fishnet metamaterial with t_Ag_=45 nm), as shown in Figure S6f. It is found that with the increase of Ag layer thickness, the maximum FOM (FOM_max_, indicating by the black dotted line) of fishnet structure increases rapidly at first, and then decreases slowly after reaching the peak (about 0.75) at the thickness of approximately 45 nm. The red dotted line represents the FOM at the position where the refractive index of the fishnet structure is the most negative, and its changing trend is consistent with the black dotted line. In addition, we also simulated and calculated the effective parameters of the fishnet structure when the thickness of the Ag layer was 0.5 and 1 nm respectively; the results showed that the refractive index was positive in the entire visible and near-infrared bands.

**S2. Preparation and characterization of the meta-cluster particles**

1. **Preparation of the meta-cluster particles**

The specific preparation process of a typical sample is as follows: 4 g of TBT is added into 30 ml of toluene and stirred for 30 min under ice bath conditions; the AgNO_3_ solution (1.5 g of silver nitrate added to 1 mL of deionized water) is added and stirred in ice bath for 30 min; 2mL of 38.5 wt% TiCl_4_ solution is added and stirred for 1 h in ice bath; the mixture is transferred to a reactor and placed in an oven, and reacted at 150°C for 24 h; after it is naturally cooled, the obtained product is washed several times with absolute ethanol, filtered and dispersed in ethanol.

The experiments show that under the condition of a certain amount of AgNO_3_ solution, the response wavelength of the ball-thorn-shaped particles gradually redshifts with the increase in the amount of TiCl_4_ solution.

1. **TiO_2_@PMMA particles**

Previously, our group has adopted solvothermal synthesis method to prepare ball-thorn-shaped TiO_2_ particles [56-58]. Here, in addition to the AgNO_3_ solution not added in the preparation of ball-thorn-shaped AgCl/TiO_2_ particles, the TiO_2_@PMMA particles are prepared according to the steps in Methods.

1. **Characterization of Ag/AgCl/TiO_2_@PMMA meta-cluster particles**

The microtopography of the particle is observed through scanning and transmission electron microscopes (Figure 2a–e, Figure 3a and b), and the local TEM image of Ag/AgCl/TiO_2_ and the corresponding electron energy loss spectroscopy (EELS) elemental mapping of Ti and Ag are taken (Figure 3c, d, and e). The crystal structure of the sample is tested using an X-ray diffractometer (Figure 2f), whereas the absorption spectrum of the sample is measured through an ultraviolet-visible-near infrared spectrophotometer (Figure 2g). The valence state and bonding situation of the Ag elements in the sample are measured the XPS spectra (Figure 3f). These studies indicate the existence of metallic Ag in the composite particles.

**S3. Preparation of 3D wedge-shaped samples and measurement of the refractive index**

1. **3D wedge-shaped samples and measurement of the wedge angles**
2. **Preparation of 3D wedge-shaped samples**

The application of the suspension of Ag/AgCl/TiO_2_@PMMA particles on the glass substrate is necessary for preparing the 3D wedge-shaped samples. To ensure that the suspension of Ag/AgCl/TiO_2_@PMMA particles can adhere well to the glass substrate during the preparation process, the glass substrate must be hydrophilically treated. The specific process of the hydrophilicity treatment of the quartz glass substrate is as follows. First, two specifications of glass strips, namely, 5 mm × 10 mm and 10 mm × 40 mm, are used. The glass strips are washed several times with scouring powder and rinsed with deionized water before transferring into a beaker. The rinsed glass strips are immersed in EtOH, and the beaker is sealed with plastic wrap. Second, the beaker is placed in an ultrasonic bath and sonicated at room temperature for 30 min. The seal is lifted off to pour off EtOH. A certain amount of deionized water is poured into the beaker to wash away the residual ethanol. Third, a mixed solution of 30% H_2_O_2_ and deionized water in a volume ratio of 7:3 is added to the beaker and boiled for 1 h at 70°C–100°C. Finally, after cooling to room temperature, the glass strips are taken out, washed with deionized water, and dried to obtain the hydrophilic quartz glass strips.

A desired suspension is prepared as follows. A suspension of the Ag/AgCl/TiO_2_@PMMA particles is flushed with a disposable plastic dropper to disperse the particles evenly. Approximately five drops of the suspension are centrifuged at the speed of 800 rpm for 30 min until the precipitate can be clearly distinguished. The supernatant is poured off, and 5 mL of deionized water is added to the suspension before performing another centrifugation under the same conditions. The supernatant is again poured off, and the precipitate of the particles is left to stand to evaporate the residual water. The deionized water with a volume 20 times of the precipitate particles is collected using a 50-μL pipette and added to the centrifuge tube to obtain the desired suspension of Ag/AgCl/TiO_2_@PMMA particles with a volume fraction of approximately 5%. Using the prepared suspension of Ag/AgCl/TiO_2_@PMMA particles, a gravity self-assembly device (Figure S7) used to prepare the 3D wedge-shaped samples is constructed. Here, the 5 mm×10 mm glass strips are customized to improve the flatness of the slope in the wedge-shaped sample. In this situation, the surface tension of the suspension will be sufficient to support the uniform gravity self-assembly of the sample. In addition, the angles on the sides of the samples can be intuitively measured. The scaling tool in Photoshop (PS) is used to scale up the side pattern of the sample to improve the accuracy of the measurement as much as possible without changing the angle.


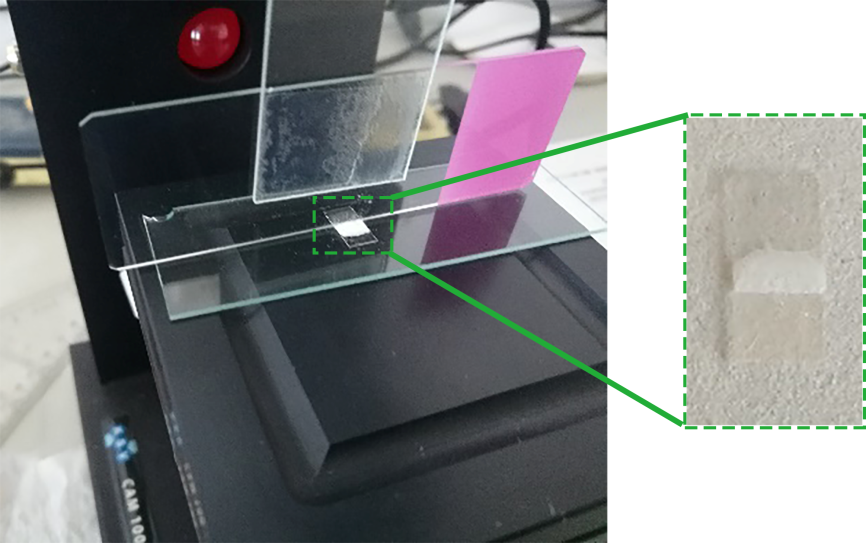


**Figure S7. Preparation of the 3D wedge-shaped samples.** A gravity self-assembly device is used to prepare the wedge-shaped sample. The right inset is the photo of the prepared wedge-shaped sample.

1. **The selection of the wedge-shaped samples**

Given that the measurement of the refractive index requires the light to pass through the sample, the wedge angle of the prepared 3D wedge-shaped sample must be as small as possible. Simultaneously, ensuring that a certain degree of deflection occurs after the light passes through the wedge-shaped sample is necessary. This procedure requires a relatively high preparation process. The slope of the wedge-shaped sample is usually not flat due to some manual errors in the preparation process. Here, the wedge-shaped samples are observed using a microscope. Considering the loss and deflection of light, the sample with relatively flat hypotenuses in the two sides of the wedge-shaped sample and with two angles between the hypotenuses and the horizontal right-angle sides between 1° and 2° is selected. Moreover, comparing the two angles to further screen out the sample with similar angles on both sides is necessary. Furthermore, the width of the sample is only 5 mm. Thus, the average of the two angles can represent the wedge angle *θ* of the sample. The side views of the selected samples are shown in Figure S8a–d. Figure S8e demonstrates the side views of the controlled sample made of TiO_2_@PMMA particles.


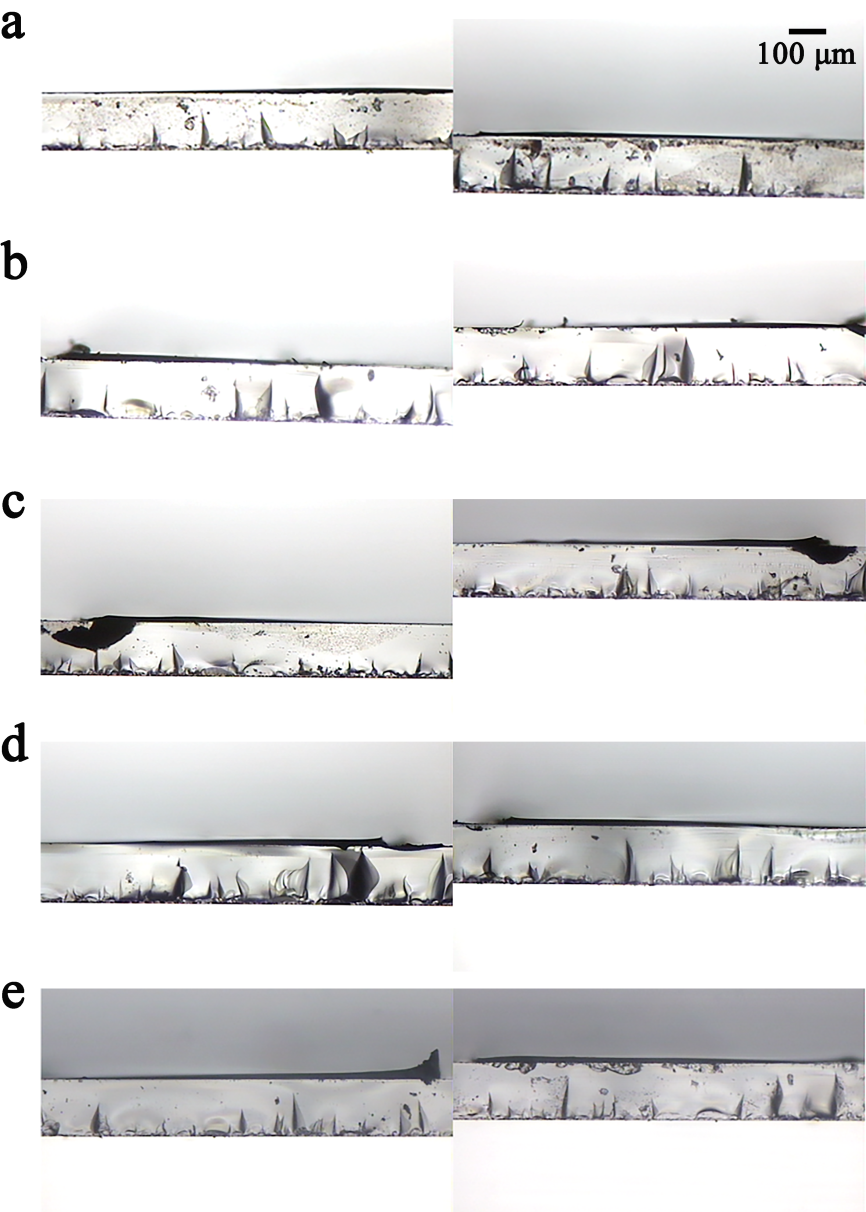


**Figure S8. Microscopic photos of the sides of the 3D wedge-shaped samples.** Side views of samples **a**, Ra and **b,** Rb responding to the red band and samples, **c,** Ga and **d,** Gb responding to the green band. **e,** Photos of the sides of a 3D wedge-shaped sample assembled by TiO_2_@PMMA particles, the angles in the two sides of this sample are 1.2° and 1.0°.

1. **The wedge angles of the red-light and green-light samples**

Figure S8 illustrates that Ra and Rb operating at the red band and Ga and Gb operating at the green band are the desired samples. The corresponding wedge angles are measured separately. The angles in the sides of samples Ra and Rb are measured using the scale tool in PS software, and then the average of the angles of each sample is calculated. The results are summarized in Table S1. In the subsequent calculation of the refractive index, the wedge angle of sample Ra is 0.8°, whereas that of sample Rb is 1.4°. Similarly, the wedge angles of samples Ga and Gb responding to the green band are measured and then also listed in Table S1. Also, in the subsequent calculation, the wedge angle of sample Ga is 1.4°, whereas that of sample Gb is 1.2°.

| Side of the wedge | *θ* (Ra) | *θ* (Rb) | *θ* (Ga) | *θ* (Gb) |
| --- | --- | --- | --- | --- |
| left | 0.8° | 1.3° | 1.5° | 1.2° |
| right | 0.8° | 1.5° | 1.3° | 1.2° |
| average | 0.8° | 1.4° | 1.4° | 1.2° |

**Table S1.** Measurement results of the wedge angles of the samples.

1. **Measurement of the refractive index**

The wedge-shaped samples formed by the gravity self-assembly device is easily damaged in a dry environment. Therefore, to avoid damaging the sample and prevent the bias of the center of the incident spot toward the edge (bottom/top edge) of the sample, the sample must be fixed on the glass substrate with four blades before measuring. The four blades are divided into two pairs that are respectively adhered to the two sides of the glass substrate in parallel. Only the slits between each pair of blades are allowed (Figure S9a). The width of the slit is between 0.5 and 1 mm, which is close to the diameter of the spot and creates the sample in the middle of the slit. The sample must also be positioned accordingly. Thus, the part of the sample with the flattest slope is in the center of the slit, and the deflection of light is the largest; such conditions are convenient for the experimental measurement. In addition, fixing the sample in this manner can reduce the influence of the surrounding stray light on the measurement results and ensure that the beam passes through the center of the sample, thereby improving the measurement accuracy.


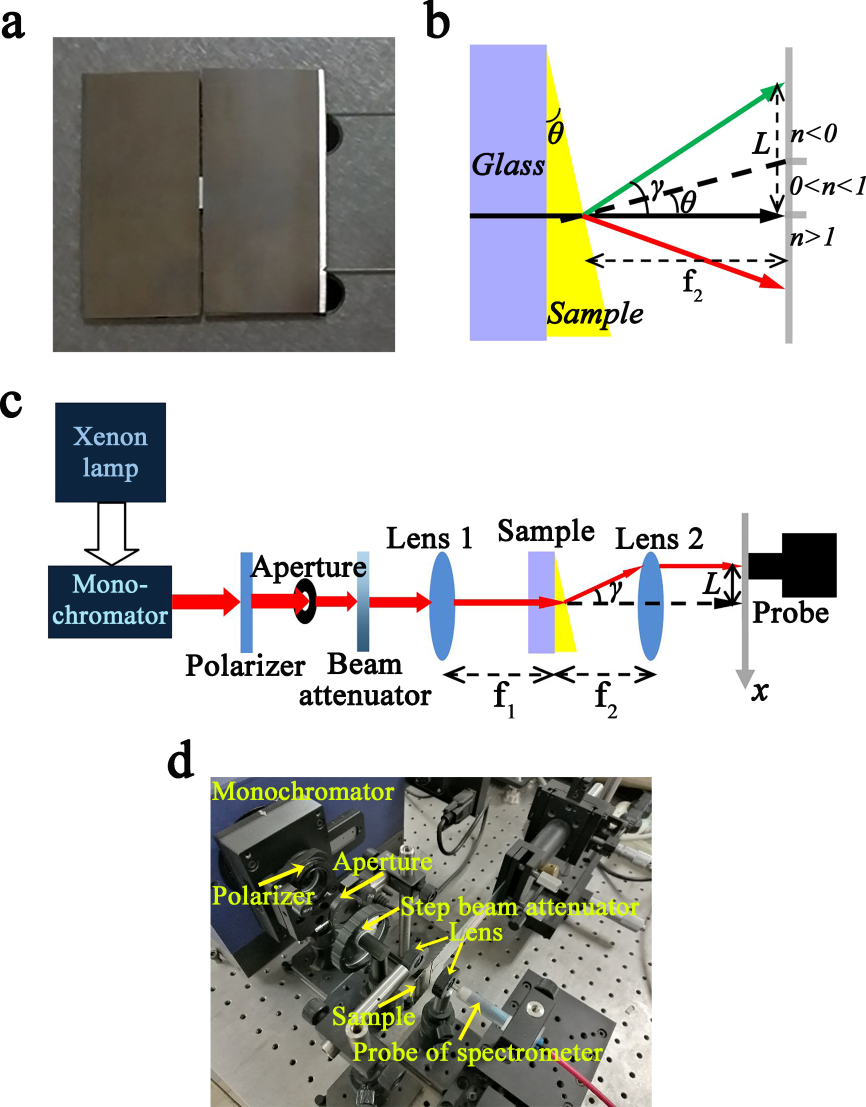


**Figure S9. Refractive index measurement for the wedge-shaped samples.** **a,** Photo of the wedge-shaped sample fixed on the glass substrate with four blades. **b,** Negative refraction measurement principle and **c,** diagram of the actual experimental setup. f_1_ = f_2_ = 12.7 mm are the focal lengths of lenses 1 and 2, respectively. The sample under test is placed at the focus of both lenses. The light beam refracted by the sample is expanded and collimated by lens 2 before being ultimately detected by a fiber spectrometer probe. This probe is installed on a micrometer displacement platform to measure the x-axis offset *L* of the refracted spot. **d,** Experimental device for refractive index measurement.

1. **Principle of measuring the refractive index**

The measurement principle [16] is illustrated in Figure S9b, and the diagram of the experimental setup is displayed in Figure S9c. A light beam is normally incident on one leg (i.e., a side adjacent to the right angle) of the wedge-shaped sample with a wedge angle of *θ*, and *γ* refers to the angle between the outgoing beam and the extension line of the incoming beam. On the receiving side, an offset *L* can be determined to quantify the refraction caused by the metamaterial sample with respect to the glass substrate, thus resulting in *γ*=tan^−1^ (*L*/f_2_), where f_2_ = 12.7 mm is the focal length of lens 2. The refractive index of the sample is then calculated in accordance with Snell’s law.

1. **Experiments of the refractive index measurement**

The experimental device is built in accordance with the experimental setup diagram displayed in Figure S9c, as depicted in Figure S9d. With a spectrometer (Ocean Optics SpectRa Suite) and a micrometer displacement platform (Zolix KSA200-12-X), the optical elements in the optical path are calibrated one by one such that their centers are on the same horizontal line. This process ensures that the center of the spot passes through the centers of these elements, thus making the measured data reliable.

The specific experimental process is as follows. The light source (ENERGETIQ LDLS TM Xenon lamp) is turned on for preheating for 20 min. The calibration is performed when the sample is not placed; thus, the light intensity is maximum when the micrometer displacement platform is at the zero position. The drop of the light intensity must be nearly the same even when the platform is adjusted from the zero position to the left or right. The fixed sample is placed on the displacement platform, and the height of the sample is adjusted such that the beam irradiates on the glass substrate without the wedge-shaped sample. At this time, the zero position must be unchanged, thereby indicating that the optical path system is collimated. Then, the height of the fixed sample is adjusted to enable the beam to irradiate the wedge-shaped sample. The wavelength of the incident light is set to a certain wavelength by adjusting the monochromator (Omni-λ 3005 No. 11072). The light intensity at different positions is detected by moving the probe. The displacement of the spot after refraction is determined by measuring the light intensity at different positions along the x-axis. Thus, the refractive index of the wedge-shaped sample with a wedge angle *θ* at this wavelength can be calculated. The moving step of the displacement platform is set to 100 units, and the size of each unit is 0.625 μm. The value of the light intensity is recorded by the probe, which is moved from the position of 1000 units to the position of −1300 units by 100 units and then moved back from the position of −1300 units to the position of 1000 units by 100 units, thus completing a cycle. Two measurement curves are obtained in a cycle. If the two curves are coincident, then the measured data are deemed available.

Each component is well-calibrated during the construction of the optical measurement platform. However, the accuracy remains inadequate because the measured wedge-shaped sample is self-assembled on the glass strip. We must also perform the last calibration of light that passes through the blank glass strip to eliminate the effect of the glass strip on the final deflection of light. In Figure S9c, a blank glass strip is used to replace a glass strip with the wedge-shaped sample. The device with the fixed blank glass strip is placed between the two lenses, and the tilt of the device is adjusted to ensure that the light is incident perpendicular to the blank glass strip. The incident wavelength is adjusted, and the deflection of the light that passes through the blank glass strip is recorded. The probe is moved in the positive and negative directions along the x-axis while measuring the light intensity at different positions to reduce accidental errors, as exhibited in Figure S10a and b. These results show that the origin of the refracted spot does not move when the light with different wavelengths is incident on the blank glass strip. Thus, the influence of the glass strip and the monochromator on the origin is negligible. The state of the optical path is debugged, and the measurement of the relevant sample can begin.


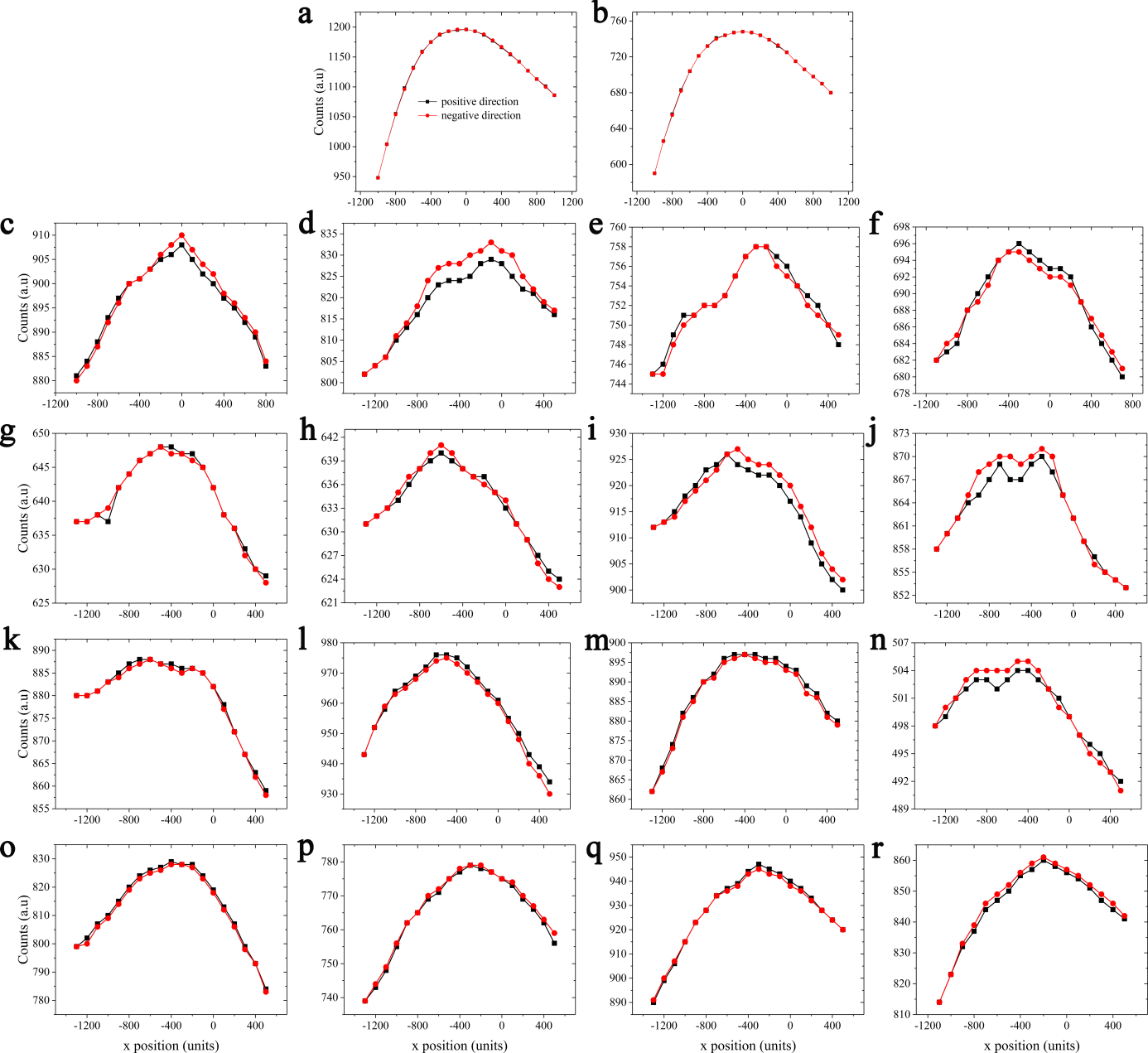


**Figure S10. Measured results of the blank glass strip and sample Rb responding to the red band.** **a,** **b** Light intensity of the refracted spot at different positions with incident wavelengths of 569.43 and 649.31 nm for the blank glass strip, respectively. **c**–**r,** Light intensity of the refracted spot at different positions with incident wavelengths of 569.57, 579.08, 589.97, 599.08, 610.24, 619.63, 624.13, 630.02, 634.82, 639.34, 644.84, 650, 655.14, 659.59, 669.15, and 679.35 nm for the sample Rb, correspondingly. The results indicated by the black curves are measured along the positive direction, and those indicated by the red curves are measured along the negative direction.

The proper samples Ra, Rb, Ga, and Gb are selected, and microscopic photos of the sides of these samples are depicted in Figure S8. Before the measurement, these samples underwent a photoreduction treatment [59, 60], irradiated by the incandescent lamp for a sufficient time (more than 5 days) to allow sufficient Ag nanoparticles to precipitate on the surface of the Ag/AgCl/TiO_2_@PMMA particles.

1. **Measurement of the red-light samples**

The measured results of sample Rb are demonstrated in Figure S10c–r. These results are obtained in accordance with the above experimental process. For other samples, only the displacement of the refracted spot and the corresponding refractive index at each incident wavelength are presented in the form of a table to avoid redundancy.

The displacement of the refracted spot at each incident wavelength is obtained using the measured results of sample Rb. The corresponding refractive index is calculated by substituting the displacement value and the wedge angle represented by the average value into the equation in the Methods. The results are listed in Table S2b. With the data in this table, the curve of the relationship between the wavelength and the refractive index is plotted in Figure S11b. The curve shows that, when the incident wavelength is in the range of 613 nm to 641 nm, sample Rb produces a negative refraction effect [53, 61, 62] with a bandwidth of 28 nm, and its refractive index reaches a maximum of −0.41 at 630 nm.

(**a**)

| Wavelength (nm) | Displace-  ment | Refractive index | Wavelength (nm) | Displace-  ment | Refractive index |
| --- | --- | --- | --- | --- | --- |
| 588.52 | 0 | 1 | 634.16 | −300 | −0.06 |
| 599.08 | −50 | 0.82 | 641.06 | −250 | 0.12 |
| 609.89 | −100 | 0.65 | 646.22 | −200 | 0.30 |
| 615.02 | −200 | 0.30 | 651.71 | −100 | 0.65 |
| 619.63 | −300 | −0.06 | 658.56 | 0 | 1 |
| 624.83 | −350 | −0.23 | 670.51 | 0 | 1 |
| 628.98 | −400 | −0.41 |  |  |  |

(**b**)

| Wavelength (nm) | Displace-  ment | Refractive index | Wavelength (nm) | Displace-  ment | Refractive index |
| --- | --- | --- | --- | --- | --- |
| 559.31 | 50 | 1.1 | 634.82 | −650 | −0.31 |
| 569.57 | 0 | 1 | 639.34 | −550 | −0.11 |
| 579.08 | −100 | 0.80 | 644.84 | −400 | 0.19 |
| 589.97 | −250 | 0.50 | 650 | −450 | 0.09 |
| 599.08 | −300 | 0.40 | 655.14 | −400 | 0.19 |
| 610.24 | −450 | 0.09 | 659.59 | −250 | 0.50 |
| 619.63 | −600 | −0.21 | 669.15 | −300 | 0.40 |
| 624.13 | −550 | −0.11 | 679.35 | −200 | 0.60 |
| 630.02 | −700 | −0.41 |  |  |  |

**Table S2.** Measured displacements of the refracted spot and calculated refractive indexes of samples (**a**) Ra and (**b**) Rb.

The displacement of the refracted spot at each incident wavelength is obtained using the measured results of sample Ra. The corresponding refractive index is calculated by substituting the displacement value and the wedge angle represented by the average value into the equation in the Methods. The results are listed in Table S2a. With these results, the curve of the relationship between the wavelength and the refractive index is shown in Figure S11a. The curve shows that when the incident wavelength is in the range of 619 nm to 636 nm, sample Ra produces a negative refraction effect with a bandwidth of 17 nm, and its refractive index reaches a maximum of −0.41 at 629 nm. A comparison of the refractive index curves of samples Ra and Rb reveals that the incident wavelengths of the maximum negative refractive response of the two samples are approximately 630 nm, and the maximum negative refractive index is approximately −0.4. The refractive index change trends of the two samples in the visible-wavelength range are also similar.


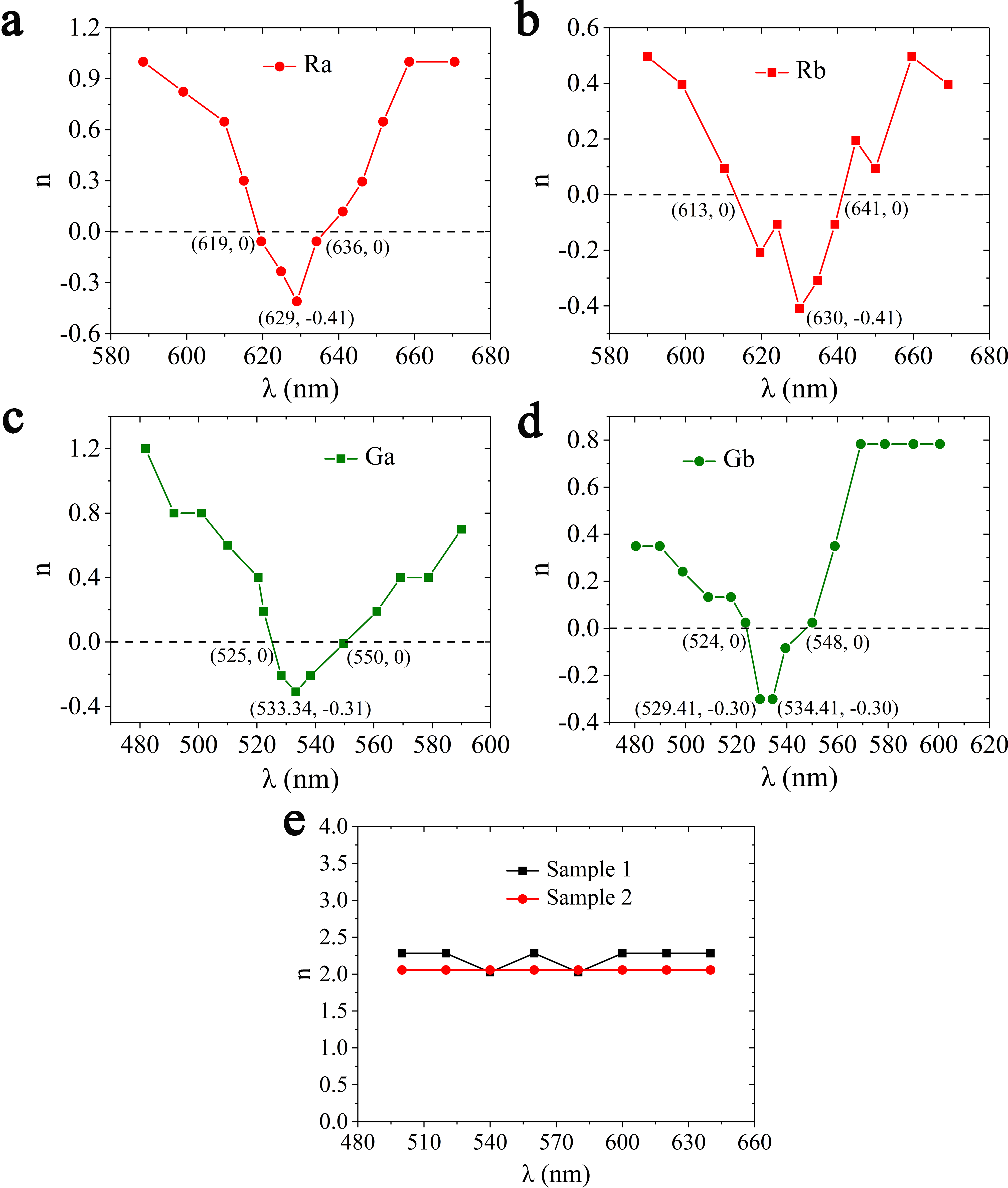


**Figure S11.** **Measurement results of refractive indexes for the wedge-shaped samples.** Refractive index curves of samples **a,** Ra and **b,** Rb and samples **c,** Ga and **d,** Gb. **e,** Refractive index curves of two samples composed of TiO_2_@PMMA particles.

1. **Measurement of the green-light samples**

The same refractive index measurement process is performed on the selected samples Ga and Gb responding to the green band. The displacement of the refracted spot at each incident wavelength is obtained using the measured results of sample Ga. The corresponding refractive index is calculated by substituting the displacement value and the wedge angle represented by the average value into the equation in the Methods. The results are listed in Table S3a. With these data, the curve of the relationship between the wavelength and the refractive index is shown in Figure S11c. The curve shows that, when the incident wavelength is in the range of 525–550 nm, sample Ga produces a negative refraction effect with a bandwidth of 25 nm, and its refractive index reaches a maximum of −0.31 at 533.34 nm.

(**a**)

| Wavelength (nm) | Displace-  ment | Refractive index | Wavelength (nm) | Displace-  ment | Refractive index |
| --- | --- | --- | --- | --- | --- |
| 481.86 | 100 | 1.20 | 533.34 | −650 | −0.31 |
| 491.64 | −100 | 0.80 | 538.34 | −600 | −0.21 |
| 501.03 | −100 | 0.80 | 549.73 | −500 | −0.01 |
| 510.04 | −200 | 0.60 | 561.08 | −400 | 0.19 |
| 520.46 | −300 | 0.40 | 569.21 | −300 | 0.40 |
| 522.34 | −400 | 0.19 | 578.73 | −300 | 0.40 |
| 528.34 | −600 | −0.21 | 589.97 | −150 | 0.70 |

(**b**)

| Wavelength (nm) | Displace-  ment | Refractive index | Wavelength (nm) | Displace-  ment | Refractive index |
| --- | --- | --- | --- | --- | --- |
| 471.33 | −200 | 0.57 | 534.41 | −600 | −0.30 |
| 480.41 | −300 | 0.35 | 539.41 | −500 | −0.08 |
| 489.83 | −300 | 0.35 | 550.09 | −450 | 0.02 |
| 498.87 | −350 | 0.24 | 558.96 | −300 | 0.35 |
| 508.96 | −400 | 0.13 | 569.21 | −100 | 0.78 |
| 517.95 | −400 | 0.13 | 578.73 | −100 | 0.78 |
| 523.68 | −450 | 0.02 | 589.97 | −100 | 0.78 |
| 529.41 | −600 | −0.30 | 600.47 | −100 | 0.78 |

**Table S3.** Measured displacements of the refracted spot and calculated refractive indexes of sample (**a**) Ga and (**b**) Gb.

Similarly, the measured data and calculation results of sample Gb are presented in Table S3b. With these data, the curve of the relationship between the wavelength and the refractive index is shown in Figure S11d. The curve shows that, when the incident wavelength is in the range of 524–548 nm, sample Gb produces a negative refraction effect with a bandwidth of 24 nm, and its refractive index reaches a maximum of −0.30 at approximately 531.42 nm. A comparison of the refractive index curves of samples Ga and Gb indicates that the incident wavelengths of the maximum negative refractive response of the two samples are nearly 532 nm. The refractive index change trends of these samples in the visible-wavelength range are also similar.

1. **Measurement of the TiO_2_@PMMA samples**

The refractive index of the sample composed of TiO_2_@PMMA particles is also measured for comparison with the abovementioned measurement results. This comparison can conveniently verify the accuracy of the optical path. It can also validate whether the negative refraction effect of the prepared samples originates from the plasmon resonance generated by the Ag nanoparticles on the particle surfaces. Two 3D wedge-shaped samples are prepared using the prepared TiO_2_@PMMA particles through the abovementioned method. The morphology of one of the two samples is displayed in Figure S8e.

The two samples are placed in the optical path, and the measured refractive index curve is plotted in Figure S11e. The average refractive index of the two TiO_2_@PMMA samples in visible light is approximately 2.1, and the fluctuation of the curves is small. Given anatase’s refractive index of 2.52, the refractive index of the TiO_2_@PMMA sample is normally smaller than 2.52 in consideration of the influence of low refractive index PMMA coating.

**S4. Measurement of the Doppler effect**

1. **Measuring system**

The inverse Doppler effect was theoretically predicted in 1968 by Veselago [10]. Indirect experimental measurements have been conducted only in nonlinear transmission lines at ~1–2 GHz in 2003 by Seddon [10], and in acoustic media at 1–3 kHz [63]. Chen, *et al.* reported the experimental observation of the inverse Doppler shift at an optical frequency (λ =10.6μm) by refracting a laser beam in a photonic-crystal prism [12]. Our group reported direct experimental measurements of the inverse Doppler effects in broadband acoustic metamaterials [64] and in pipe instruments [65]. Since the existing technology cannot directly detect the frequency of visible light, it is necessary to use the heterodyne interferometry [12] to indirectly measure the Doppler shift. According to the method in ref. [12], we designed a high-precision laser heterodyne detection system based on the refraction of a visible laser beam through the prepared metamaterial wedge-shaped sample [66]. As shown in Figure S12, the polarized light emitted from the semiconductor laser is split into two beams by the beam splitter (BS) 1, and one of the two beams passing through the sample is used as the measurement beam and normally incident on a right-angle plane of the wedge-shaped sample. The measurement beam is refracted at the interface between the sample and air. The other beam is used as the reference beam, which reaches the BS 2 after being reflected by two reflectors. The reference and the measurement beams meet at the BS 2 and produce interference. The final interference beam signal focused by the lens is collected by the detector (Dsi200). During the measurement, the wedge-shaped sample and BS 2 are fixed together on the electronically translation stage (KSA200-12-X); the stage can do uniform linear motion with velocity $v$ under the control of computer. The direction of motion in the experiment is set to the direction away from the detector. The BS 2 moves when the stage moves, so the Doppler shift also occurs in the measurement beam. Due to the different amount of produced frequency shift in the measurement beam and the reference beam, the final frequency of the two beams is different, and “light beat” occurs after interference. After the detector receives this beat frequency signal, the frequency difference between the two beams can be obtained by fast Fourier transform.


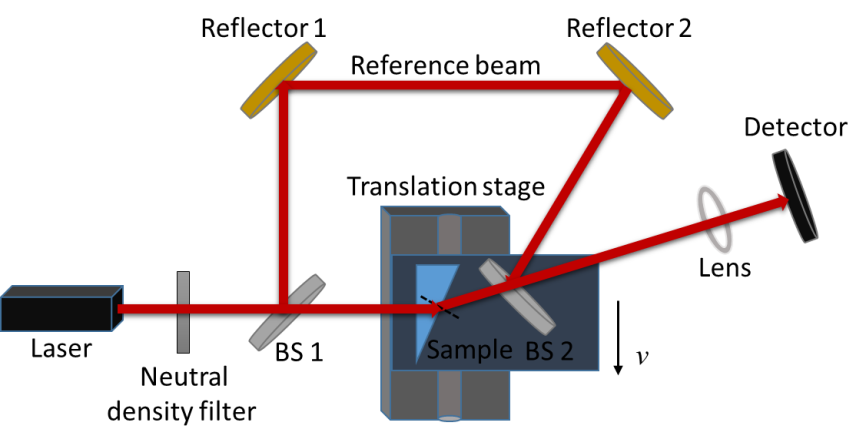


**Figure S12.** Schematic of the heterodyne interference measuring system to measure the Doppler effect.

When the sample moves, the change of the measuring optical path is shown in Figure S13. The solid and dashed lines indicate the optical path before and after the sample moves respectively. The process of relative motion is decomposed into two steps: first, when the sample moves with the stage along the direction of the arrow, the process of increasing the optical path in the sample can be equivalent to extending the exit position (moving from point O to point O’) while the position where the light is incident on the sample is unchanged. Therefore, for the light propagating in the sample, it is equivalent to that the supposed “observer” at the exit position has moved away, which causes the first Doppler shift; for the refracted beam, the point of refraction has a displacement at the interface between the sample and the air, which can be regarded as a light source moving in the direction toward the detector. As a consequence, the measurement beam will have a second Doppler shift in the air, and the final frequency of measurement beam is the result of the superposition of the two Doppler shifts. The derivation of the Doppler shift generated during the entire motion is as follows.


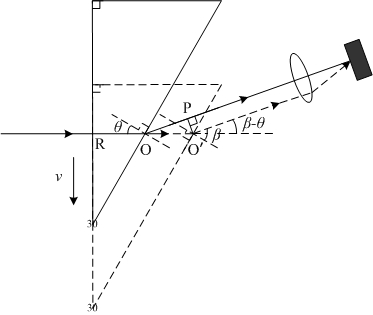


**Figure S13.** Schematic of the measurement optical path when the wedge-shaped sample moves.

Because the sample moves at the velocity $v$ along the direction of the arrow, the velocity component of the exit light point (that is, the supposed “observer”) on the sample in the direction ($\overrightarrow{OO'}$) of light propagation can be written as

, (6)

where *θ* is the wedge angle of the sample. Assuming that the original frequency of the laser is$f_{0}$, the speed of light in vacuum is *c*, and the refractive index of the sample is *n*, thus the frequency of light passing through the sample at the exit position is

. (7)

Since the sample moves while the detector remains stationary, the measurement beam at the refraction point can be regarded as a light source moving toward the detector. So the measurement beam undergoes a second Doppler shift. The refraction angle is assumed to be *β*, and the velocity component of the refraction point (that is, the imaginary “light source”) for the measurement beam in the direction ($\overrightarrow{OP}$) of the exiting light is

. (8)

Finally, when the measurement beam reaches the detector, the theoretical value of its frequency is

$f_{2}=f_{1}\left(\frac{c}{c-v_{2}}\right)=f_{0}\left(1-\frac{v_{1}}{c}n\right)\left(\frac{c}{c-v_{2}}\right)$. (9)

Because the BS 2 moves with the translation stage, the reference beam also has a Doppler shift. The optical path of the reference beam changes as the BS 2 moves, as shown in Figure S14. As in Figure S13, the solid and dashed lines represent the optical paths before and after the movement of the BS 2, respectively.


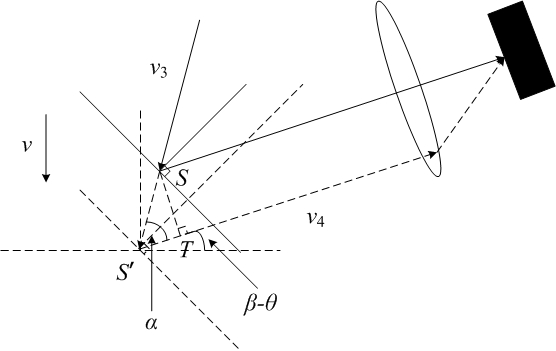


**Figure S14.** Schematic of the reference optical path when the BS 2 moves.

Assuming the angle between the incident light and the reflected light is , then the velocity component of the BS 2 in the incident direction of the reference beam is

$v_{3}=\frac{\cos\left(\pi/2-\alpha/2-\beta+\theta\right)}{\cos\left(\alpha/2\right)}v$. (10)

When the reference beam is incident on the BS 2, its frequency is

$f'_{1}=f_{0}\left(1-\frac{v_{3}}{c}\right)$. (11)

Then, the light reflected from the BS 2 is regarded as the “light source” of the reflected beam. The velocity component of the BS 2 along the direction of the reflected beam is

. (12)

Finally, when the reference beam reaches the detector, the theoretical value of its frequency is

. (13)

Therefore, the frequency difference between the measurement beam and the reference beam is

. (14)

Let , so the equation (9) can be written as

$f_{2}=f_{1}k$. (15)

Substituting the formula (15) into the equation (14) obtains,

$\Delta f=\left|f'_{2}-f_{2}\right|=\left|\left(f'_{2}-f_{0}k\right)-\left(f_{1}-f_{0}\right)k\right|$. (16)

where $\left(f_{1}-f_{0}\right)$ is the Doppler shift that occurs within the sample, and *k* is always positive and approaches 1 in a non-relativistic ($v<<c$) system. In the equation (16), the first part on the right side of the equation is an independent part, which has nothing to do with the positive or negative refractive index of the sample and only depends on the angles in the experiment. Using the measuring system designed here, the final frequency difference $\Delta f$ between the measurement and the reference beams can be measured. Since the first part can be calculated from the detailed experimental conditions, the value of $\left(f_{1}-f_{0}\right)k$ can be obtained by using the measured beat frequency$\Delta f$. Therefore, the measured $\Delta f$ can be used to determine the amount of Doppler shift inside the sample.

1. **Improved measuring system**

After preliminary analysis and some experimental tests, we improve the heterodyne interference measuring system by fixing the BS 2 on the optical platform based on the above measuring system and without affecting the function of the measuring system, as shown in Figure S15. After the improvement, the Doppler shift of the measurement beam will only occur when the stage is moving, and the reference beam will be in a relatively static state, and no frequency change will occur. The measured beat frequency is equal to the frequency difference between the measurement and the reference beams.


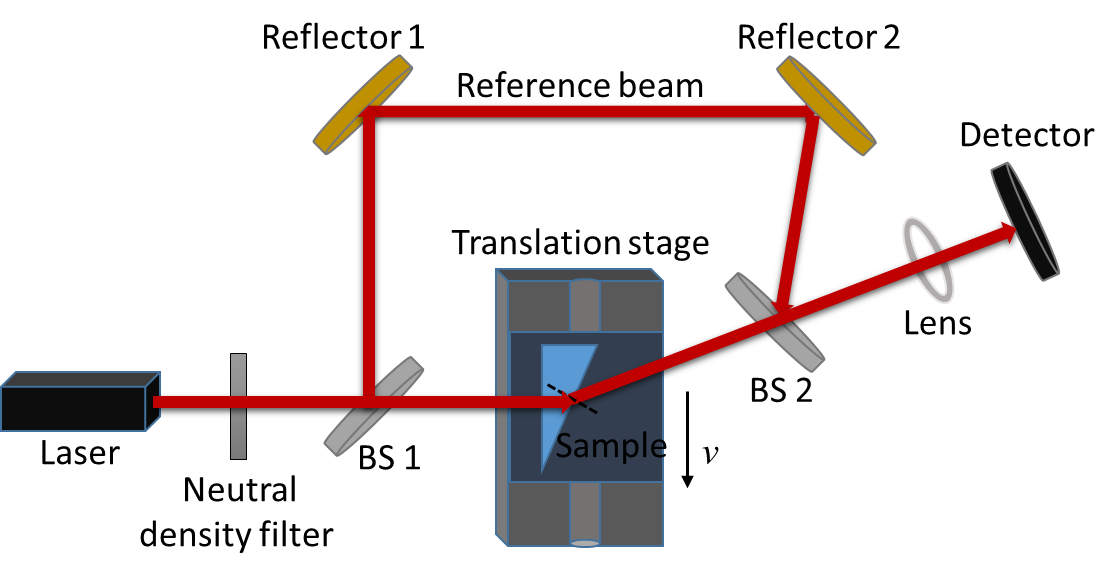


**Figure S15.** Schematic of the improved heterodyne interference measuring system to measure the Doppler effect.

When the stage moves, the propagation of the measurement beam in the sample (assumed normal material) is still shown in Figure S13. The theoretical derivation of the frequency shift for the measurement beam is the same as described above. Finally, when the measurement beam reaches the detector, its theoretical frequency is

. (17)

Because there is no frequency shift here and the original frequency is maintained for the reference beam, so the theoretical value of the beat frequency generated by the two beams is

. (18)

Substituting equations (7) and (17) into the equation (18) gives

. (19)

Thus, we get the theoretical value of the measured beat frequency through the detector after the improvement. In order to distinguish between normal Doppler shift and inverse Doppler shift, some transformations of the equation (18) are needed.

Set $k=\frac{c}{c-v\tan\theta\cdot\cos\left(\beta-\theta\right)}$, then the equation (17) can be transformed into

$f_{2}=f_{1}k$. (20)

Substitute the equation (20) into the equation (18) and transform the equation (18) into

, (21)

where $\left(f_{1}-f_{0}\right)$ is the Doppler shift that occurs inside the sample. $(k-1)f_{0}$ is calculated according to the experimental conditions, and the value of $\left(f_{1}-f_{0}\right)k$ can be obtained by using the measured beat frequency $\Delta f$. In this way, the Doppler shift of the light inside the sample is retrieved. If the Doppler shift in the sample is negative (that is, ), it means that the frequency of the light becomes smaller when the measurement beam exits from the sample during the process of being away, and the red shift phenomenon occurs; the normal Doppler effect occurs inside the sample, indicating the sample is a normal material. Conversely, if is positive (that is,$f_{1}>f_{0}$), the blue shift phenomenon occurs during the process of being away; the inverse Doppler effect occurs inside the sample, indicating the sample is a metamaterial.

1. **Verification of the improved measuring system through positive index materials**
2. **Doppler effect of the K9 crystal prism**

In order to verify the feasibility of the improved measuring system, the K9 crystal prism is first used as a sample for the Doppler effect measurement. First each optical element is adjusted to the same height and coaxial, and the angle of the reflector is finely tuned so that the measurement beam and the reference beam converge to the BS 2. The convex lens is placed in front of the detector so that the detector is at its focal position. And by fine-tuning the angle of the BS 2, the measurement beam and the reference beam finally enter the center of the detector so as to completely coincide, and interference fringes are generated. The incident beam is always perpendicular to the photosensitive surface of the detector to ensure that when the wedge-shaped sample moves, the measurement and the reference beams can still focus and superpose on the detector. In the experiment, when the stage carries the sample together and moves at a uniform speed, the interfering beam produces a beat frequency signal. Using the oscilloscope connected to the computer and the Lab VIEW Signal Express software on the computer, the beat frequency signal received by the detector is displayed. After fast Fourier transform processing in the software, the beat frequency signal is converted into a power spectrum density map, thereby calculating the Doppler shift that occurs inside the sample.


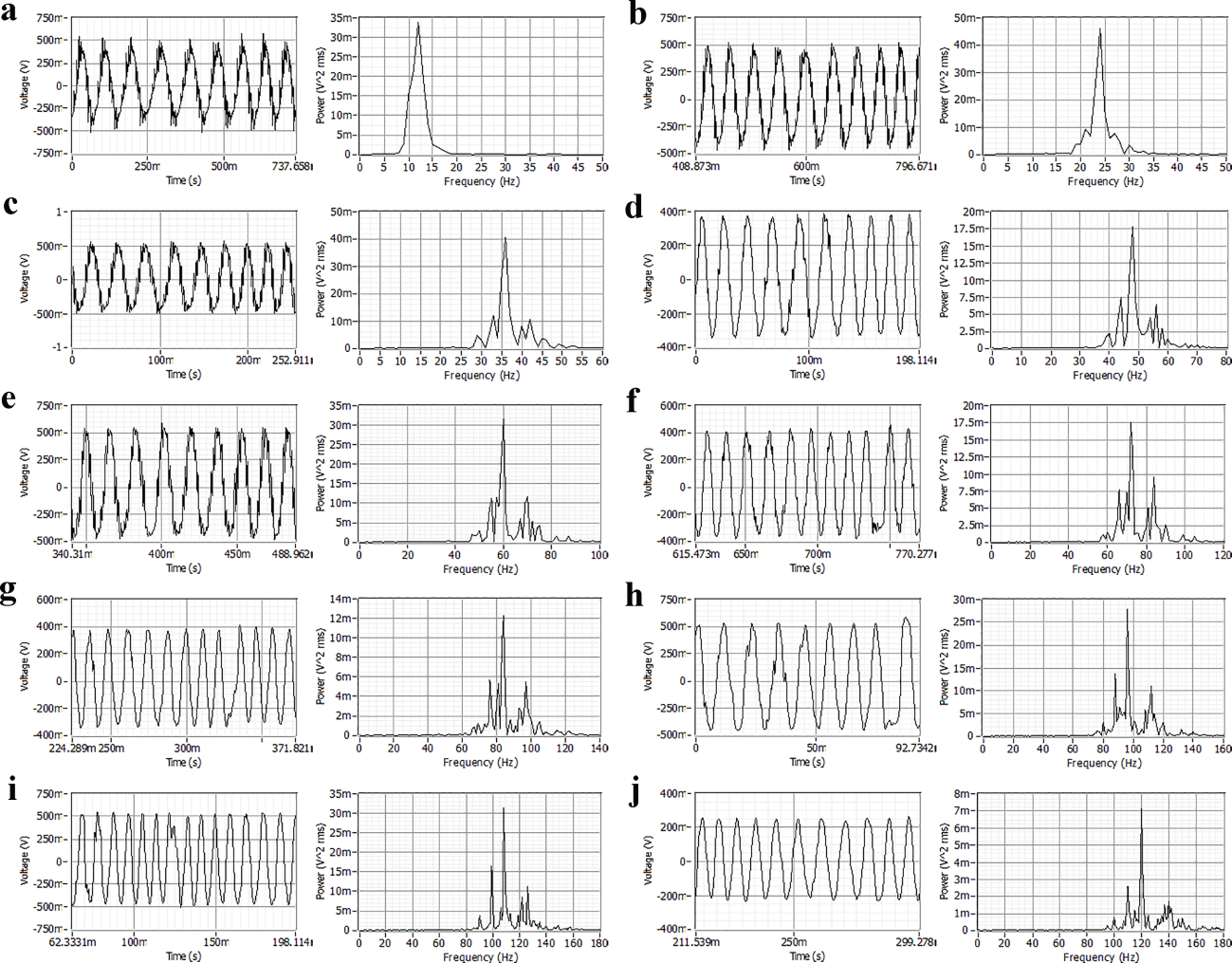


**Figure S16.** Waveform map and power spectrum density map of the beat frequency signal obtained at different velocities. **a,** *v* =20 μm/s. **b,** *v* =40 μm/s. **c,** *v* =60 μm/s. **d,** *v* =80 μm/s. **e,** *v* =100 μm/s. **f,** *v* =120 μm/s. **g,** *v* =140 μm/s. **h,** *v* =160 μm/s. **i,** *v* =180 μm/s. **j,** *v* =200 μm/s.

A semiconductor laser with a wavelength of 532 nm is used as the light source in the experiment, and the refractive index of the K9 crystal prism for 532 nm light is 1.5195. The incident angle *θ* = 30°, and the refraction angle *β* = 49.44°. In the experiment, we used 20 μm/s as the velocity gradient and measured the Doppler effect of the electronically translation stage moving at 10 different velocities from 20 μm/s to 200 μm/s. The waveform map and power spectrum density map of the beat frequency signal obtained at different velocities are shown in Figure S16. The theoretical and measured values of the Doppler shift experiments are listed in Table S4, and the relative error of the beat frequency is calculated. Finally, the beat frequency $\Delta f$ of the K9 crystal at different velocities and the Doppler shift $\left(f_{1}-f_{0}\right)k$ in the measured sample are shown in Figure S17.

| *v* (μm/s) | $(k-1)f_{0}$ (Hz) | Theoretical (Hz) | Measured (Hz) | Relative measurement error of (%) | Theoretical (Hz) | Measured (Hz) |
| --- | --- | --- | --- | --- | --- | --- |
| 20 | 20.468 | 12.513 | 12 | 4.099 | -32.981 | -32.468 |
| 40 | 40.935 | 25.026 | 24 | 4.099 | -65.961 | -64.935 |
| 60 | 61.403 | 37.539 | 36 | 4.099 | -98.942 | -97.403 |
| 80 | 81.870 | 50.052 | 48 | 4.099 | -131.922 | -129.870 |
| 100 | 102.338 | 62.565 | 60 | 4.099 | -164.903 | -162.338 |
| 120 | 122.805 | 75.078 | 72 | 4.099 | -197.883 | -194.805 |
| 140 | 143.273 | 87.591 | 84 | 4.099 | -230.864 | -227.273 |
| 160 | 163.741 | 100.104 | 96 | 4.099 | -263.845 | -259.741 |
| 180 | 184.208 | 112.617 | 108 | 4.099 | -296.825 | -292.208 |
| 200 | 204.675 | 125.130 | 120 | 4.099 | -329.805 | -324.675 |

**Table S4.** Measured results of the K9 crystal prism.


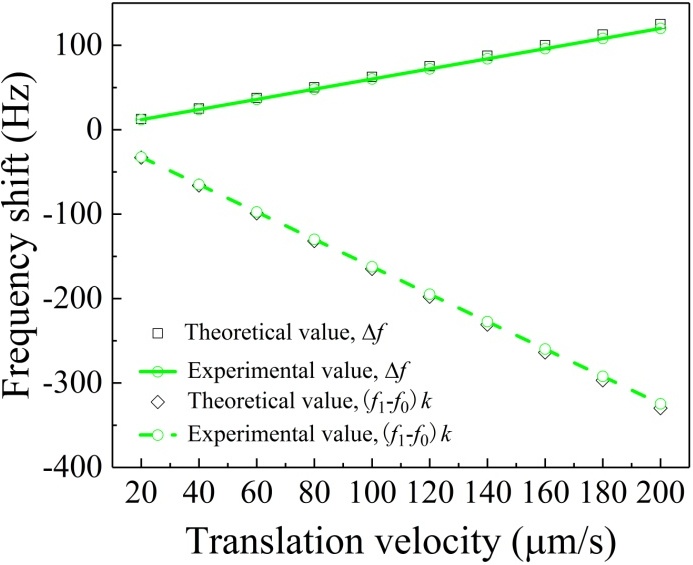


**Figure S17.** Beat frequency $\Delta f$ and Doppler shift $\left(f_{1}-f_{0}\right)k$ in the K9 crystal at different velocities.

Figure S16 shows the waveform maps of the beat frequency signals for the K9 crystal at different movement velocities and the corresponding beat frequencies obtained through the fast Fourier transform of the signals recorded by the detector in the measurement software. It is found that the values of the measured beat frequency are 12, 24, 36, 48, 60, 72, 84, 96, 108, and 120 Hz, respectively. Obviously, the value of the beat frequency increases linearly with the increase of velocity, which is consistent with the theoretical prediction of the beat frequency. Through calculation, it is found that the value of measured beat frequency is very close to that of the theoretical prediction, and the relative error is very stable and less than 5%. It can be found from Figure S17 that the experimental and theoretical values of the beat frequency and Doppler shift are very close, and the experimental and theoretical values of these two sets of curves are highly consistent. At these velocities, the measured Doppler shift inside the sample is all negative, that is, the frequency $f_{1}$ after the Doppler shift occurs is less than the original frequency $f_{0}$ of the light source. Combined with experimental analysis, when the optical path becomes longer, the frequency of the outgoing light decreases, indicating a red-shift phenomenon, and a normal Doppler effect occurs. The measurement result accords with the theoretical prediction, which verifies the accuracy of the measuring system well.

1. **Doppler effect of the TiO_2_@PMMA wedge-shaped sample**

The final sample used for the measurement is a 3D wedge-shaped sample assembled by Ag/AgCl/TiO_2_@PMMA particles, which has a large difference from the size and morphology of the K9 crystal. For excluding the influence of other factors in the measurement, a wedge-shaped sample made of TiO_2_@PMMA particles is used to conduct a verification experiment. It is worth mentioning that the sample used for Doppler effect measurement must have sufficient width (at least 2 mm) to ensure that it can be moved on the stage.

A semiconductor laser with a wavelength of 532 nm is also used as the light source, and the wedge angle of the sample is *θ* = 1.8°. According to experimental measurements, the refractive index of the TiO_2_@PMMA wedge-shaped sample is n = 2.1, so the refraction angle *β* = 3.782°. The measurement conditions are the same as the experiment of the K9 crystal. The data measured at 10 different velocities are shown in Table S5, and the beat frequency and the Doppler shift results are shown in Figure 5d in the text.

| *v* (μm/s) | $(k-1)f_{0}$ (Hz) | Theoretical (Hz) | Measured (Hz) | Relative measurement error of (%) | Theoretical (Hz) | Measured (Hz) |
| --- | --- | --- | --- | --- | --- | --- |
| 20 | 1.181 | 1.300 | 1.2 | 7.713 | -2.481 | -2.381 |
| 40 | 2.361 | 2.601 | 2.4 | 7.713 | -4.962 | -4.761 |
| 60 | 3.542 | 3.901 | 4 | 2.541 | -7.443 | -7.542 |
| 80 | 4.723 | 5.201 | 5.2 | 0.022 | -9.924 | -9.923 |
| 100 | 5.904 | 6.501 | 6.4 | 1.560 | -12.405 | -12.304 |
| 120 | 7.084 | 7.802 | 7.6 | 2.586 | -14.886 | -14.684 |
| 140 | 8.265 | 9.102 | 9.2 | 1.076 | -17.367 | -17.465 |
| 160 | 9.446 | 10.402 | 10.4 | 0.022 | -19.848 | -19.846 |
| 180 | 10.627 | 11.703 | 11.4 | 2.586 | -22.329 | -22.027 |
| 200 | 11.807 | 13.003 | 13.2 | 1.516 | -24.810 | -25.007 |

**Table S5.** Measured results of the TiO_2_@PMMA wedge-shaped sample.

As shown in Figure 5d, the deviation between the experimental value and the theoretical value of the beat frequency is small, and the two curves basically coincide. It can also be seen intuitively that the values of $\left(f_{1}-f_{0}\right)k$ are all negative. According to the previous analysis, the value of the Doppler shift in the sample is negative during the process of being away, that is, the red shift phenomenon occurs, so the normal Doppler effect is generated. These results are consistent with theory.

Through the above two experimental measurements, not only the correctness of the measuring system is verified, but also the sensitivity of this system to the wedge-shaped sample with a small wedge angle is fully proved, leading to achieve an accurate measurement.

1. **Measurement of inverse Doppler effect of metamaterials in the visible band**

According to the completed negative refraction experiment, we measured the inverse Doppler effect for the two samples with known refractive index. Due to the requirement for sample width in the refractive Doppler effect measurement, we prepared two wedge-shaped samples assembled by ball-thorn shaped Ag/AgCl/TiO_2_@PMMA metamaterial with transmission peaks at 525 and 640 nm respectively, which are denoted as samples Gc (responding to green light) and Rc (responding to red light) respectively. And the Doppler effect of the two wedge-shaped samples (Gc and Rc) is measured respectively. The difference of the wedge angles of the two selected wedge-shaped samples is small, and the wedge angles of the samples Rc and Gc are *θ* = 1.4° and 1.7°, respectively.

1. **Theoretical derivation of measurement of the inverse Doppler effect**

Since the wedge-shaped sample is composed of negative refractive index metamaterials, the measurement beam will undergo negative refraction when it exits from the sample to the air, and the measurement optical path is shown in Figure 5a. In this measuring system, when the stage moves in a specified direction, the propagation of the measurement beam in the sample is shown in Figure S18.


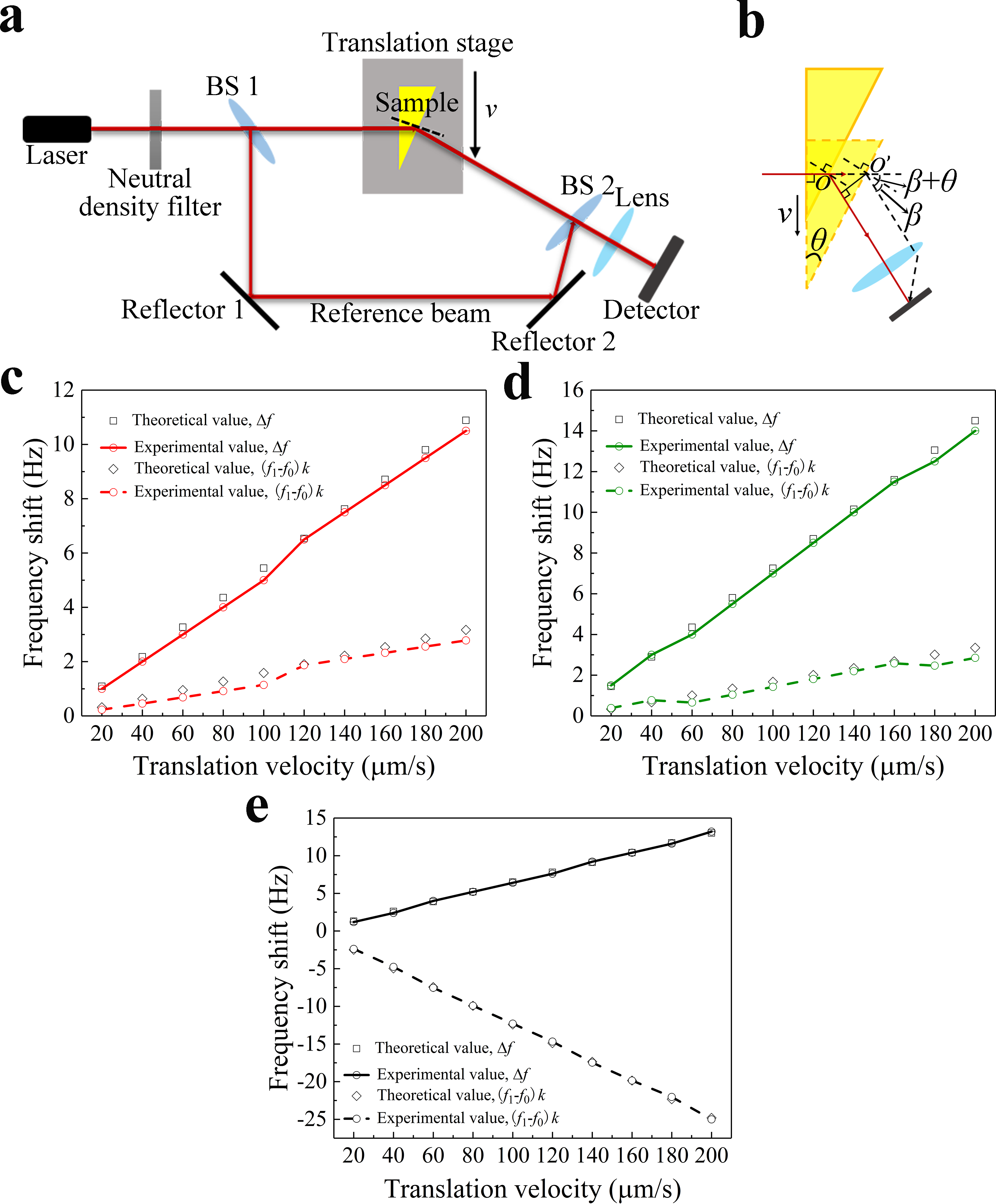


**Figure S18.** Schematic of the reference optical path when the stage moves.

Assuming the refractive index of the metamaterial wedge-shaped sample is *n_p_*, the velocity of the stage is *v*, the frequency of incident laser is$f_{0}$, and the corresponding wavelength is $\lambda$, thus the first Doppler shift that occurs inside the sample is

$f_{1}=f_{0}\text{(1}-\frac{v\tan\theta}{c}n_{p}\text{)}$, (22)

and the second Doppler shift that occurs outside the sample is

$\begin{matrix} f_{2}=f_{1}\frac{c}{c-v\tan\theta\cdot\cos\left(\theta+\beta\right)} \\ =f_{0}\left[1+\frac{v}{c}\left(\tan\theta\cdot\cos\left(\theta+\beta\right)-\tan\theta\cdot n_{p}\right)\right] \end{matrix}$. (23)

Because the reference beam has no frequency shift, the beat frequency detected by the detector is

$\begin{matrix} \Delta f=\left|f_{2}-f_{0}\right| \\ =\left|f_{0}\cdot\frac{v}{c}\left(\tan\theta\cdot\cos\left(\theta+\beta\right)-\tan\theta\cdot n_{p}\right)\right| \end{matrix}$. (24)

Because $n_{p}=-\frac{\sin\beta}{\sin\theta}$, the above equation can be simplified as

$\Delta f=\frac{v}{\lambda}\sin\left(\theta+\beta\right)$. (25)

Let $k=\frac{c}{c-v\tan\theta\cdot\cos\left(\theta+\beta\right)}$, then the equation (23) can be expressed as

$f_{2}=f_{1}k$. (26)

Substituting the equation (26) into the equation (24) gives

, (27)

where $(k-1)f_{0}$ can be transformed into

$(\text{k}-1)\text{f}_{0}=\frac{\text{v}\tan\theta\cdot\cos(\theta+\beta)}{\text{c}-\text{v}\tan\theta\cdot\cos(\theta+\beta)}\text{f}_{0}=\frac{\text{v}}{\lambda}\tan\theta\cdot\cos(\theta+\beta)$, (28)

and $\left(f_{1}-f_{0}\right)$ is the Doppler shift of the measurement beam generated inside the sample.

Similarly, the measured beat frequency $\Delta f$ can be used to infer the Doppler shift of the measurement beam inside the wedge-shaped sample. If the Doppler shift in the sample is negative (that is, ), it means that the frequency of the light becomes smaller when the measurement beam exits from the sample during the process of being away, and the red shift phenomenon occurs; the normal Doppler effect occurs inside the sample, indicating the sample is a normal material. Conversely, if is positive (that is,$f_{1}>f_{0}$), the blue shift phenomenon occurs during the process of being away; the inverse Doppler effect occurs inside the sample, indicating the sample is a metamaterial.

1. **Measurement of the inverse Doppler effect for the red-light sample Rc**

A semiconductor laser with a wavelength of 632.8 nm is used to measure the sample Rc (wedge angle *θ* = 1.4°, refractive index n = -0.41), and the waveform and frequency spectrum of the beat frequency signal at different velocities are obtained. The measurement data are shown in Table S6. The beat frequency and Doppler shift of the red-light sample at different velocities are shown in Figure 5b.

| *v* (μm/s) | $(k-1)f_{0}$ (Hz) | Theoretical (Hz) | Measured (Hz) | Relative measurement error of (%) | Theoretical (Hz) | Measured (Hz) |
| --- | --- | --- | --- | --- | --- | --- |
| 20 | 0.772 | 1.089 | 1.000 | 8.173 | 0.317 | 0.228 |
| 40 | 1.544 | 2.177 | 2.000 | 8.130 | 0.633 | 0.456 |
| 60 | 2.316 | 3.266 | 3.000 | 8.145 | 0.950 | 0.684 |
| 80 | 3.088 | 4.355 | 4.000 | 8.152 | 1.267 | 0.912 |
| 100 | 3.860 | 5.443 | 5.000 | 8.139 | 1.583 | 1.140 |
| 120 | 4.632 | 6.532 | 6.500 | 0.490 | 1.900 | 1.868 |
| 140 | 5.404 | 7.621 | 7.500 | 1.588 | 2.217 | 2.096 |
| 160 | 6.176 | 8.709 | 8.500 | 2.400 | 2.534 | 2.324 |
| 180 | 6.948 | 9.798 | 9.500 | 3.041 | 2.850 | 2.552 |
| 200 | 7.720 | 10.887 | 10.500 | 3.555 | 3.167 | 2.780 |

**Table S6.** Measured results of the red-light sample Rc.

It is found from these results that the measured beat frequency agrees with the theoretical value; when the velocity is lower than 100 μm/s, the relative error is some large, but the slope of the curve is relatively stable, the coincidence between the theoretical value and the experimental value is very high, and the absolute error is very small, indicating the results is credible. We believe that because of the influence of the velocity accuracy of the stage, the relative error is some large when the velocity is relatively small. The calculated Doppler shifts of the sample Rc at different velocities are all positive, that is, the frequency of the outgoing light increases when the optical path becomes longer, so an inverse Doppler effect occurs inside the sample.

1. **Measurement of the inverse Doppler effect for the green-light sample Gc**

As with the above experimental method, a semiconductor laser with a wavelength of 532 nm is used to measure the sample Gc (wedge angle *θ* = 1.7°, refractive index n = -0.3). The measured data and calculated results are shown in Table S7 and Figure 5c.

| *v* (μm/s) | $(k-1)f_{0}$ (Hz) | Theoretical (Hz) | Measured (Hz) | Relative measurement error of (%) | Theoretical (Hz) | Measured (Hz) |
| --- | --- | --- | --- | --- | --- | --- |
| 20 | 1.115 | 1.450 | 1.500 | 3.448 | 0.335 | 0.385 |
| 40 | 2.230 | 2.899 | 3.000 | 3.484 | 0.669 | 0.770 |
| 60 | 3.345 | 4.349 | 4.000 | 8.025 | 1.004 | 0.655 |
| 80 | 4.460 | 5.799 | 5.500 | 5.156 | 1.339 | 1.040 |
| 100 | 5.575 | 7.248 | 7.000 | 3.422 | 1.674 | 1.425 |
| 120 | 6.690 | 8.698 | 8.500 | 2.276 | 2.008 | 1.810 |
| 140 | 7.805 | 10.148 | 10.000 | 1.458 | 2.343 | 2.195 |
| 160 | 8.919 | 11.597 | 11.500 | 0.836 | 2.678 | 2.581 |
| 180 | 10.034 | 13.047 | 12.500 | 4.193 | 3.013 | 2.466 |
| 200 | 11.149 | 14.497 | 14.000 | 3.428 | 3.347 | 2.851 |

**Table S7**. Measured results of the green-light sample Gc.

Similar to the measured results of red-light sample, the measured beat frequency for the green-light sample is very close to the theoretical value; the relative errors as a whole are small and reasonable except for a slight fluctuation at 60 μm/s, demonstrating the results are reliable. It is found through calculation that all the obtained Doppler shifts of the green-light sample are positive, indicating that the frequency of the outgoing light increases and a blue shift occurs during the process of being away, so an inverse Doppler effect is observed.

**Supplementary References**

[56] J. Yin, X. Zhao, L. Xiang, X. Xia, Z. Zhang.“Enhanced electrorheology of suspensions containing sea-urchin-like hierarchical Cr-doped titania particles,” *Soft Matter*, vol. 5, p. 4687, 2009.

[57] L. Xiang, X. Zhao, J. Yin, B. Fan.“Well-organized 3D urchin-like hierarchical TiO 2 microspheres with high photocatalytic activity,” *J. Mater. Sci.*, vol. 47, p. 1436-1445, 2012.

[58] L. Xiang, X. Zhao, C. Shang, J. Yin.“Au or Ag nanoparticle-decorated 3D urchin-like TiO_2_ nanostructures: synthesis, characterization, and enhanced photocatalytic activity,” *J. Colloid Interf. Sci.*, vol. 403, p. 22-8, 2013.

[59] Y. A. Wu, L. Li, Z. Li, et al.,“Visualizing Redox Dynamics of a Single Ag/AgCl Heterogeneous Nanocatalyst at Atomic Resolution,” *ACS nano*, vol. 10, p. 3738-3746, 2016.

[60] R. Dong, B. Tian, C. Zeng, T. Li, T. Wang, J. Zhang.“Ecofriendly Synthesis and Photocatalytic Activity of Uniform Cubic Ag@AgCl Plasmonic Photocatalyst,” *J. Phys. Chem. C*, vol. 117, p. 213-220, 2013.

[61] D. R. Smith, N. Kroll.“Negative refractive index in left-handed materials,” *Phys. Rev. Lett.*, vol. 85, p. 2933, 2000.

[62] V. M. Shalaev, W. Cai, U. K. Chettiar, et al.,“Negative index of refraction in optical metamaterials,” *Opt. Lett.*, vol. 30, p. 3356-3358, 2005.

[63] X. Hu, Z. Hang, J. Li, J. Zi, C. T. Chan.“Anomalous Doppler effects in phononic band gaps,” *Phys. Rev. E*, vol. 73, p. 015602, 2006.

[64] S. L. Zhai, X. P. Zhao, S. Liu, F. L. Shen, L. L. Li, C. R. Luo.“Inverse Doppler Effects in Broadband Acoustic Metamaterials,” *Sci. Rep.*, vol. 6, p. 32388, 2016.

[65] S. L. Zhai, J. Zhao, F. L. Shen, L. L. Li, X. P. Zhao.“Inverse Doppler Effects in Pipe Instruments,” *Sci. Rep.*, vol. 8, p., 2018.

[66] S. Zhang, Y. Hu, X. Zhao.“On the Refractive Laser Doppler Velocimetry,” *J. Experimental Mechanics*, vol. 28, p. 409-415, 2013.
